# Single-Trial Recognition of Imagined Forces and Speeds of Hand Clenching Based on Brain Topography and Brain Network

**DOI:** 10.1007/s10548-018-00696-3

**Published:** 2018-12-31

**Authors:** Xin Xiong, Yunfa Fu, Jian Chen, Lijun Liu, Xiabing Zhang

**Affiliations:** School of Automation and Information Engineering, Kunming University of Science and Technology, Kunming, 650500 People’s Republic of China

**Keywords:** Brain-computer interfaces, Microstate, Topography, Brain network, Imagined force and speed of hand clenching

## Abstract

To provide optional force and speed control parameters for brain–computer interfaces (BCIs), an effective feature extraction method of imagined force and speed of hand clenching based on electroencephalography (EEG) was explored. Twenty subjects were recruited to participate in the experiment. They were instructed to perform three different actual/imagined hand clenching force tasks (4 kg, 10 kg, and 16 kg) and three different hand clenching speed tasks (0.5 Hz, 1 Hz, and 2 Hz). Topographical maps parameters and brain network parameters of EEG were calculated as new features of imagined force and speed of hand clenching, which were classified by three classifiers: linear discrimination analysis, extreme learning machines and support vector machines. Topographical maps parameters were better for recognition of the hand clenching force task, while brain network parameters were better for recognition of the hand clenching speed task. After a combination of five types of features (energy, power spectrum of the autoregressive model, wavelet packet coefficients, topographical maps parameters and brain network parameters), the recognition rate of the hand clenching force task was 97%, and that of the hand clenching speed task was as high as 100%. The brain topographical and the brain network parameters are expected to improve the accuracy of decoding the EEG signal of imagined force and speed of hand clenching. A more efficient brain network may facilitate the recognition of force/speed of hand clenching. Combined features could significantly improve the single-trial recognition rate of imagined forces and speeds of hand clenching. The current study provides a new idea for the imagined force and speed of hand clenching that offers more control intention instructions for BCIs.

**Electronic supplementary material:**

The online version of this article (10.1007/s10548-018-00696-3) contains supplementary material, which is available to authorized users.

## Introduction

Brain-computer interfaces (BCIs) are a revolutionary human–computer interaction (Remsik et al. 2016; Zhang et al. 2016; Ahn and Jun 2015) that are expected to provide potential communication and control applications for specific patients or specific scenes (Baykara et al. 2016; Yin et al. 2015a, b; Ang et al. 2015). At present, the practical technology of BCIs still needs to narrow the gap between research and the real world.

The motor imagery BCIs is an important aspect of the BCIs paradigm (He et al. 2015), which is driven by the implicit psychological activity of the subjects, in which an EEG signal is readily detectable in healthy (Yuan and He 2014), as well as disabled, individuals with neuromuscular diseases or injuries, including spinal-cord injury, amyotrophic lateral sclerosis (ALS), and stroke (He et al. 2013). Many efforts have been devoted to using BCIs to interface with physical devices by bypassing the neuromuscular pathways, including virtual helicopters (Doud et al. 2011), physical quadcopters (LaFleur 2013), wheelchairs (Tanaka et al. 2005; Carlson and Millan 2013a) and telepresence robots (Carlson et al. 2013b). These BCIs have the potential to restore lost or impaired functions of people severely disabled by various devastating neuromuscular disorders or spinal-cord damage and to enhance or supplement functions in healthy individuals (He et al. 2015).

Many studies have demonstrated that the neural activity before or during exercise encodes direction, speed, and other information about movement (Aschersleben 2002; Kopp et al. 2000; Gerloff et al. 1998; Gu et al. 2009a, b, c; Nascimento and Farina 2008; Farina et al. 2007; Yuan and He 2014). So far, researchers have proposed many methods to extract EEG patterns of motor imagery, such as Energy (Gu et al. 2009a; Li et al. 2004; Fu et al. 2017; Yin et al. 2015a, b; Sun et al. 2015), Hilbert–Huang transform (Fu et al. 2017; Yin et al. 2015a, b; Sun et al. 2015), Autoregression (AR), Adaptive autoregression (AAR) (Schlögl et al. 1997; D’Croz-Baron et al. 2012; Yom-Tov and Inbar 2002), Wavelet transform (WT), Wavelet package transform (WPT) (Zhou et al. 2012, Hsu 2010; Farina et al. 2007), Common spatial pattern (CSP) (Yang and Hu 2013), EEG source imaging (ESI) (He et al. 2015; Edelman et al. 2016), entropy (Wang et al. 2012), EEG microstate (Biasucci et al. 2011; Pirondini et al. 2017; Minguillon et al. 2014) and some other methods (Yom-Tov and Inbar 2002; Jochumsen et al. 2013).

Based on these features, certain classification accuracies are obtained, but the accuracy and stability of recognition need to be improved greatly for the actual application. The functional states of the brain are constantly changing, and the EEG signal has a high time resolution, which enables it to detect the instantaneous states of the brain. Microstate analysis is one of the methods of analyzing the instantaneous states of the brain, which defines the states of the multichannel EEG signals by spatial topographies of electric potentials over the electrode array (Lehmann et al. 1987). When the EEG signal is considered as a time series of topographies, there are two remarkable properties. First, although there are a large number of topographies of an EEG signal, the majority of signals can be represented by few maps. Second, a single topography remains dominant for approximately 80–120 ms before abruptly transitioning to another topography. These periods of quasi-stability of a single topography are microstates. Thus, the multichannel EEG signals could be represented by a series of microstates at discrete intervals (Khanna et al. 2014).

At present, there are more studies on the microstate analysis of resting-state EEG (Khanna et al. 2015), such as behavioral states (Lehmann et al. 2010), personality types (Schlegel et al. 2012), neuropsychiatric disorders (Kikuchi et al. 2011), sleep classes (Brodbeck et al. 2012), and perceptual awareness (Britz et al. 2014). However, there are few studies on the microstate analysis of task-state EEG, such as auditory stimulation (Ott et al. 2011) and visual stimulation (Antonova et al. 2015). Studies on the motor imagery task for BCI are much fewer, such as executing motor imagery of the affected and unaffected hands of stroke patients’ (Biasucci et al. 2011) pure planar reaching movements as well as reaching and grasping of different objects (Pirondini et al. 2017; Minguillon et al. 2014). There is scant research on the microstate analysis of actual/imagined hand clenching force/speed based on EEG, and there is even scanter research on the further application of the single-trial recognition of hand clenching force and speed. Therefore, this study used topographical maps parameters to identify the hand clenching force and speed of the single trial.

In addition to the above, functional differentiation and integration of the human brain are the two major organizational principles of human brain function (Liang et al. 2010). Although different regions of the brain have relatively different functions, completing a very simple task also requires interaction and mutual coordination of multiple different functional regions, and these brain regions together constitute a complete network; that is, the execution of the brain function always depends on extensive interaction of multiple brain regions. The complex network analysis based on graph theory, an effective method for studying neural connections or functional connections between the brain regions which has greatly promoted the understanding of the human brain network’s organization pattern, is an areas of intense research in neuroscience (Liang et al. 2010; Jiang et al. 2009; Zhang et al. 2015a, b; Zhang et al. 2013; Xu et al. 2014; Laufs et al. 2012; Li et al. 2016). “Node” and “edge” are two important concepts in graph theory. Therefore, the two steps of building the brain network are as follows: definition of network nodes and network connections. For EEG recording, the electrodes are used as network nodes, and various measures, such as correlation, synchronization likelihood, and coherence, can be used to calculate their functional connectivity (Rubinov and Sporns 2010; Vinck et al. 2011; Qin et al. 2010; van den Heuvel et al. 2009). Partial directed coherence (PDC) analysis is often used to research the directional relations between multichannel time series, which was put forward by Baccalá and Sameshima (2001) to describe the directed relationship between the multivariate time series. Many researchers have constructed EEG networks using PDC for post-stroke depression (Wang et al. 2015), cognitive load (Chen et al. 2015), seizure (Gang et al. 2016), and somatosensory vibration (Ma et al. 2016). These studies inspired us to adopt a brain network to analyze actual/imagined hand clenching force/speed based on EEG. The functional connection between the brain regions, which reflects the changes in brain activity and the integration of function during the execution or motor imagery period, may be probably employed to identify the force/speed of hand clenching.

Aiming at the single-trial recognition of the actual/imagined force and speed of hand clenching, we for the first time tried to use the topographical maps parameters and the brain network parameters as new characteristics of EEG to improve the recognition accuracy. In addition, the traditional features (energy, power spectrum of AR and wavelet packet coefficient) were also extracted for a comparative study. After constructing the EEG eigenvectors, three classifiers of LDA, extreme learning machines (ELMs) and SVM were used to identify the new feature vectors. The current study was expected to provide an additional force-control and speed-control intention instruction for the motor imagery BCI system and to provide some inspiration for the realization of more advanced brain-control robots.

## Materials and Methods

### Subjects

Twenty healthy subjects (12 males and 8 females, with an average age of 22.8 ± 5.1 years and an undergraduate and master’s degree) were enrolled in EEG data acquisition. All subjects were right-handed and had no history of sensorimotor impairment or mental illness that affected brain function. Before the experiment, they gave informed consent for the study, which was approved by the Research Ethics Board of the Kunming University of Science and Technology, and were given a motor imagery aptitude test questionnaire.

## Experimental Protocol

**Fig. 1:**
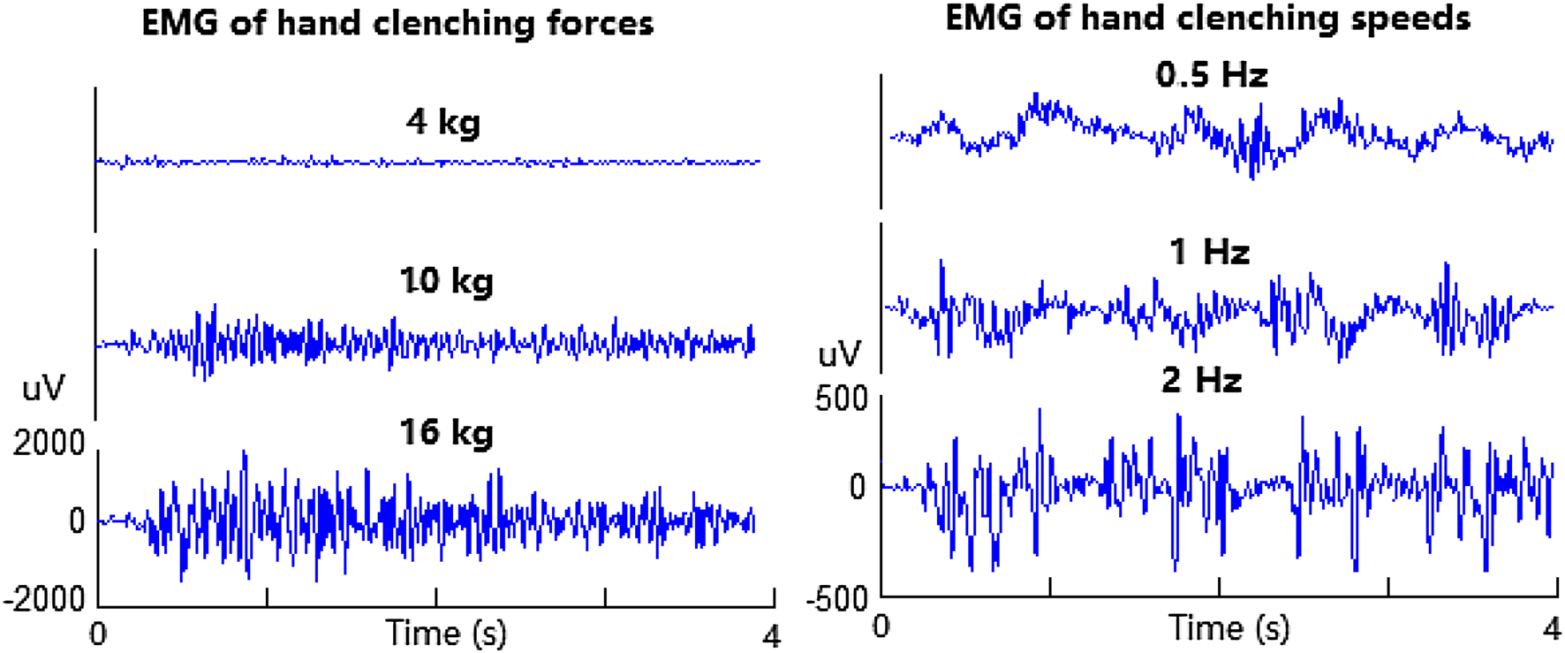
EMG of hand clenching forces at 4 kg, 10 kg, and 16 kg and speeds at 0.5 Hz, 1 Hz, and 2 Hz

For the hand clenching force task, the subjects were instructed to execute/imagine three different forces (4 kg, 10 kg and 16 kg) involving the right hand, measured by a grip force scale for executing hand clenching forces during the training and the formal EEG signals acquisition. For the hand clenching speed task, the subjects were instructed to execute/imagine three different speeds (0.5 Hz, 1 Hz and 2 Hz) involving the right hand using a comfortable force, paced by metronome during the training and the formal EEG signals acquisition. During the experiment, myoelectricity (EMG) was collected simultaneously to reflect the changes of executing hand clenching force/speed, illustrated in Fig. 1, in which the EMG lasted one second at rest, seven seconds at executed force/speed period, and there were no recordings at imagined task. EMG electrode was placed at the ventral side of forearm approximately five centimeters from the wrist.

**Fig. 2:**
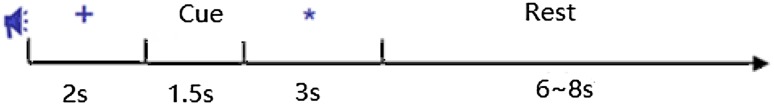
Timing schematic diagram of executed/imagined force/speed of hand clenching

During the experiment, the subjects were seated in a comfortable chair and asked to remain calm. Each subject at each force or speed task participated in three sessions, and each session involved 30 trials (10 trials for 4 kg, 10 kg and 16 kg forces respectively or 10 trials for 0.5 Hz, 1 Hz and 2 Hz speeds respectively). The imagined tasks followed executed tasks. Figure 2 illustrates the timing diagram of a single trial for executed/imagined force/speed of hand clenching.

A beep sound indicated the beginning of each trial, and simultaneously a cross (+) was displayed in the center of the screen for 2 s, during which the subjects were asked to remain relaxed and be ready for the trial. Then, a cue in the form of a picture appeared on the white screen indicating the force of a hand clenching performance/imagination of 4/10/16 kg or a speed of 0.5/1/2 Hz. The sequence of forces/speeds was randomized. The cue lasted 1.5 s, and the subjects were instructed to get ready for actual/imagined hand clenching force/speed. The subjects began to execute the cued task when the cued picture disappeared from the screen and a black star-shaped fixation cursor appeared on the white screen. The task was maintained for a period of 3 s until the fixation cursor disappeared from the screen, and during the period no online identification results were provided for the subject. When the task ended, the screen went blank, and the subject was given a 6–8 s rest before the next trial.

A trial lasted 10.5–12.5 s, and the total time of each session lasted 5.25–6.25 min. The experiment consisted of three sessions with a 10 min break between them. The entire experiment was finished within 1 h, including the preparation time. During the trials, subjects were asked to avoid blinking, slow eye movement, the activities of facial muscle and other body parts except for the random rest interval between trials.

Subjects were trained about the imagined task every day for 1 h, and the training lasted 2 weeks before data acquisition. The subjects were instructed to perform a kinematic imagery of the hand movement, rather than visual imagery (i.e. recalling or feeling themselves clenching a hand with different forces and speeds at the first personal perspective, rather than mentally watching them or another person executing the task). Substantial training was conducted to enhance the compliance of the subjects until the researchers verified that subjects performed the movement appropriately and until the subjects reported vivid imagery of the task in a questionnaire on motor imagery ability.

A total of nine EEG electrodes over the primary motor area and the supplementary motor area were used in this research (FC3, FCz, FC4, C3, Cz, C4, CP3, Pz, and CP4). The EEG recording was referenced to the bilateral mastoid (M1 and M2) and grounded at electrode Fpz. Electrodes were made of a Ag–AgCl powder. The EEG signals were acquired by Neuroscan Synamps 2 at a sampling frequency of 1000 Hz. The electrode impedance was reduced to 5000 Ω before the experiment. The electro-oculogram (EOG) was also recorded to ensure that no EOG artifact existed during the motor imagery task’s period (Fu et al. 2015).

## Data Processing and Feature Extraction

### Data Preprocessing

The EEG signals were pre-processed with the EEGLAB toolbox for MATLAB, which were re-referenced to the common average reference, high-pass filtered with a 0.05 Hz zero-phase FIR filter to remove offset and trend, low-pass filtered with a 48 Hz zero-phase FIR filter, and down-sampled to 125 Hz. And the EEG signals were inspected for artifacts with a procedure based on Independent Components (ICs) using ADJUST plug-in (Brunner et al. 2013): IC scalp maps and frequency spectra were inspected, and ICs that displayed features indicative of artifacts were removed (Mognon et al. 2011).

### Feature Extraction

#### Topographical Maps Parameters

Topographical maps derive from microstate analysis. The microstate analysis of EEG is carried out as follows: first, the global field power (GFP) of *K* electrodes is calculated, and the local maximum values of GFP are obtained, which represent instants of strongest field strength and highest topographical signal-to-noise ratio; second, the peaks of GFP are employed to generate topographical maps of the electrode array, and the topographic maps are grouped into clusters; finally, the parameters of each microstate cluster are calculated.

The calculation formula of GFP is as follows (Khanna et al. 2014):

1 $$\text{GFP}=\sqrt{\left({\sum }_{\text{i}}^{\text{k}}{\left({V}_{i}\left(t\right)-{V}_{mean}\left(t\right)\right)}^{2}\right)/k}$$

where ${V}_{i}\left(t\right)$ represents the instantaneous electric field of electrode *i*, ${V}_{mean}\left(t\right)$ represents the average instantaneous electric field of all the *K* electrodes. GFP reflects the electric field intensity at each *t* moment of the brain, which is typically used to measure the response of the brain to an event or to describe the rapid changes of brain activity.

To obtain representative microstates clusters, all topological maps corresponding to local maximum values of GFP had to be clustered. The optimal number of clusters was indicated by cross validation (Pascualmarqui et al. 1995; Koenig et al. 2014).

The parameters of the microstates offer a variety of quantifications of the EEG signals with potential neurophysiological relevance (Khanna et al. 2015). The *duration* of a microstate reflects the stability of its underlying neural assemblies. The *occurrence* of a microstate reflects the tendency of its underlying neural generators to become activated. The *time coverage* of a microstate reflects the relative time coverage of its underlying neural generators compared to others. The *amplitude* of a microstate reflects the strength or degree of the neurons in underlying neural generators. The four parameters are computed as follows:

- *Duration* of a microstate is the average time of duration during which a given microstate remained stable whenever it appears.
- *Occurrence* of a microstate is the average number of occurrence per second that the microstate becomes dominant during the recording period.
- *Occurrence* of a microstate is the average number of occurrence per second that the microstate becomes dominant during the recording period.
- *Amplitude* of a microstate is the average GFP during the microstate dominance.

In the current study, *duration, occurrence, time coverage*, and *amplitude* of topographical maps were employed for recognition, and the number of topographical maps parameters is presented in Table 1.

**Table 1:** The number of five types of features for recognition

Types of features	Topographical maps parameters	Brain network parameters	Energy	Power spectrum of the AR model	Wavelet packet coefficients
Number of features	3 × 4	2 × 3	3 × 9	3 × 9 × 125	4 × 9

#### Brain Network Parameters

In the current study, partial directed coherence (PDC) (Baccalá et al. 2001) was used to measure connectivity of the brain’s functional network. The PDC formulas are as follows:

2 $$\mathbf{A}\left(f\right)=\sum _{i=1}^{p}{C}_{i}{e}^{-j2\pi if/{f}_{s}}$$

3 $$\overline{A} \left( f \right) = \user2{I} - \user2{A}\left( \user2{f} \right) = \user2{I} - \mathop \sum \limits_{{i = 1}}^{p} C_{i} e^{{ - j2\pi if/f_{s} }} 0$$

4 $$PDC_{{x_{j} \to x_{k} }} \left( \user2{f} \right) = \frac{{\overline{{a_{{k,j}} }} (f)}}{{\sqrt {\mathop \sum \nolimits_{{i = 1}}^{m} |\overline{{a_{{i,j}} }} (f)|^{2} } }}$$

where ${C}_{i}$is the AR model coefficient of the EEG signals $\text{X}\left({x}_{1},{ x}_{2}, \dots , {x}_{n}\right)$of *N* leads in the time domain, $\overline{{a_{{k,j}} }} \left( f \right)$ is the *i*th element of the *j*th column in $\overline{A}\left(f\right)$. The value range of ${PDC}_{{x}_{j}\to {x}_{k}}$ is [0, 1], representing the proportion of the signals flowing from ${x}_{j}$ to ${x}_{k}$ to that of all signals flowing from ${x}_{j}$; the one close to 0 indicates that there is no connection between the two channels, and the one close to 1 indicates that the two channels are strongly linked, and the intensity of information flow is high.

In the current study, nine electrodes were used as network nodes, and the information flow intensity calculated by PDC was used as the edge of the network. For the generated brain network, two topological parameters, *clustering coefficient* and *shortest path length*, were quantitatively described.

*Clustering coefficient* measures the degree of collectivization of a network, indicating that the neighbors of node *i* could become neighbors. The *clustering coefficient C*_*i*_ of a node *i* is the ratio of the number of edges (*e*_*i*_) in the node’s neighbors and the number of possible edges ($\frac{{k}_{i}({k}_{i}-1)}{2}$), as follows:

5 $${C}_{i}=\frac{2{e}_{i}}{{k}_{i}({k}_{i}-1)}$$

Due to a large number of nodes in complex network, the average clustering coefficient of all the nodes from the perspective of statistics rather than clustering coefficient of each node is researched, i.e.,

6 $$\text{C}= <{C}_{i}> =\frac{1}{N}\sum _{i\in V}{C}_{i}$$

*Shortest path length* depicts the optimal path from a node’s information to another node in the network, indicating that the information passing through the shortest path transfers faster and thereby system resources are saved. The path of least edges between two nodes *i* and *j* is called the shortest path between the two nodes, and the number of edges passing through the path is the shortest path length *l*_*ij*_ between nodes *i* and *j*.

In the research on complex networks, the shortest path length between each two nodes is seldom investigated, but the average shortest path length of the whole network is usually investigated, as follows:

7 $$\text{L}=\frac{2}{N(N-1)}\sum _{i,j\in V,i\ne j}{l}_{ij}$$

In the current study, the *clustering coefficient* and the *shortest path length* of Theta (4–8 Hz), Alpha (8–13 Hz) and Beta (13–30 Hz) bands were employed for recognition, and the number of brain network parameters is presented in Table 1.

#### Traditional Methods of Feature Extraction

To carry out the comparative study in this study, the following traditional feature-extraction methods were also employed:

##### Energy

The ERD/ERS phenomenon of EEG provides a scientific basis for BCIs based on motor imagery. Energy is one of the most common features of EEG; in this paper, the energy formula is provided in Eq. (8) (Pfurtscheller and Lopes 1999):

8 $${E}_{n}\left(\%\right)=\frac{{e}_{n}-{e}_{c}}{{e}_{c}}\times 100$$

where *e*_*c*_ is the average energy of the reference idling period, and *e*_*n*_ is the average energy of executed/imagined force/speed of hand clenching.

In this study, *e*_*c*_ was the average energy of 1 s before 0 instant (the cued picture disappeared), and *e*_*n*_ was calculated every second during the executed/imagined force/speed of a hand clenching period lasting 3 s. Because of nine electrodes, the number of energy features for recognition was 3 × 9, as presented in Table 1.

##### Power Spectrum of Auto-regressive (AR) Model

Power spectrum is also one of the common features of EEG. Compared with the traditional power spectrum analysis method, the power spectrum estimation of the AR model only need short-range data to obtain spectral estimation with a higher resolution, and it could be easily transformed into feature vectors (Zhou and Luo 2013).

The formula of power spectrum estimation of the AR model is obtained:

9 $${\text{P}}\left( \omega \right) = \sigma ^{2} /\left( {\left| {1 + \sum\limits_{{i = 1}}^{p} {c_{{pi}} } e^{{ - j\omega }} } \right|} \right)$$

where *p* is the order of the AR model, *c*_*pi*_ is the undetermined weight parameter, and ${\sigma }^{2}$is the variance of the white noise residual.

To avoid the inconsistent intensity of EEG signals causing unstable eigenvalues, which the subjects produced when they performed the same kind of hand clenching tasks at different times, the normalized power spectrums of the AR model corresponding to the Mu rhythm (8–13 Hz) and the Beta rhythm (13–30 Hz) were used (Zhou and Luo 2012):

10 $$\text{P}=\frac{\left[\sum _{\omega =8}^{13}P\left(\omega \right)+\sum _{\omega =13}^{30}P\left(\omega \right)\right]}{\sum P\left(\omega \right)}$$

Because the power spectrums of the AR model were calculated in the frequency domain, with the resampled rate of 125 Hz, the length of signal for every second after FFT transformation was 125 points. On account of nine electrodes and the executed/imagined force/speed of hand clenching period lasting 3 s, the number of power spectrums of the AR model for recognition was 3 × 9 × 125, illustrated in Table 1.

##### Wavelet Packet Coefficients

Wavelet packet is suitable for the analysis of non-stationary EEG signals, which could continue to decompose the *W* space and improve the accuracy of signal analysis. The wavelet packet decomposition algorithm for discrete signals is as follows (Morlet et al. 1982):

11 $$\left\{\begin{array}{c}{d}_{l}\left(j,2n\right)=\sum _{k}{a}_{k-2l}{d}_{k}(j+1,n)\\ {d}_{l}\left(j,2n+1\right)=\sum _{k}{b}_{k-2l}{d}_{k}(j+1,n)\end{array}\right.$$

where *a*_*k*_, *b*_*k*_ are the conjugate filter coefficients of wavelet packet decomposition.

In the current study, the EEG signals were carried on a three-layer wavelet packet decomposition process, in which the frequency band of the node *d* (*3, 1*) was near the Alpha spectrum (8–13 Hz) and the frequency band of the nodes *d* (*3, 2*), *d* (*3, 3*) as well as *d* (*3, 4*) were near the Beta spectrum (13–30 Hz) (Xu et al. 2011), while the ERD/ERS phenomenon of motor imagery EEG mainly appears in these two bands (Pfurtscheller and Lopes 1999). Therefore, the wavelet packet coefficients of these four nodes were extracted as features for recognition. Because of nine electrodes, the number of wavelet packet coefficients was 4 × 9, as presented in Table 1.

#### Classifier

In the current study, for the hand clenching force task, actual and imagined tasks were classified into three classes (executed/imagined forces of 4 kg, 10 kg, and 16 kg); similarly, for the hand clenching speed task, actual/imagined hand clenching speed tasks were also classified into three classes (executed/imagined speeds of 0.5 Hz, 1 Hz, and 2 Hz), and LDA, SVM as well as ELMs (Huang et al. 2012) were used as classifiers.

## Results

### Topographical Maps Parameters of Actual and Imagined Forces and Speeds of Hand Clenching

**Fig. 3:**
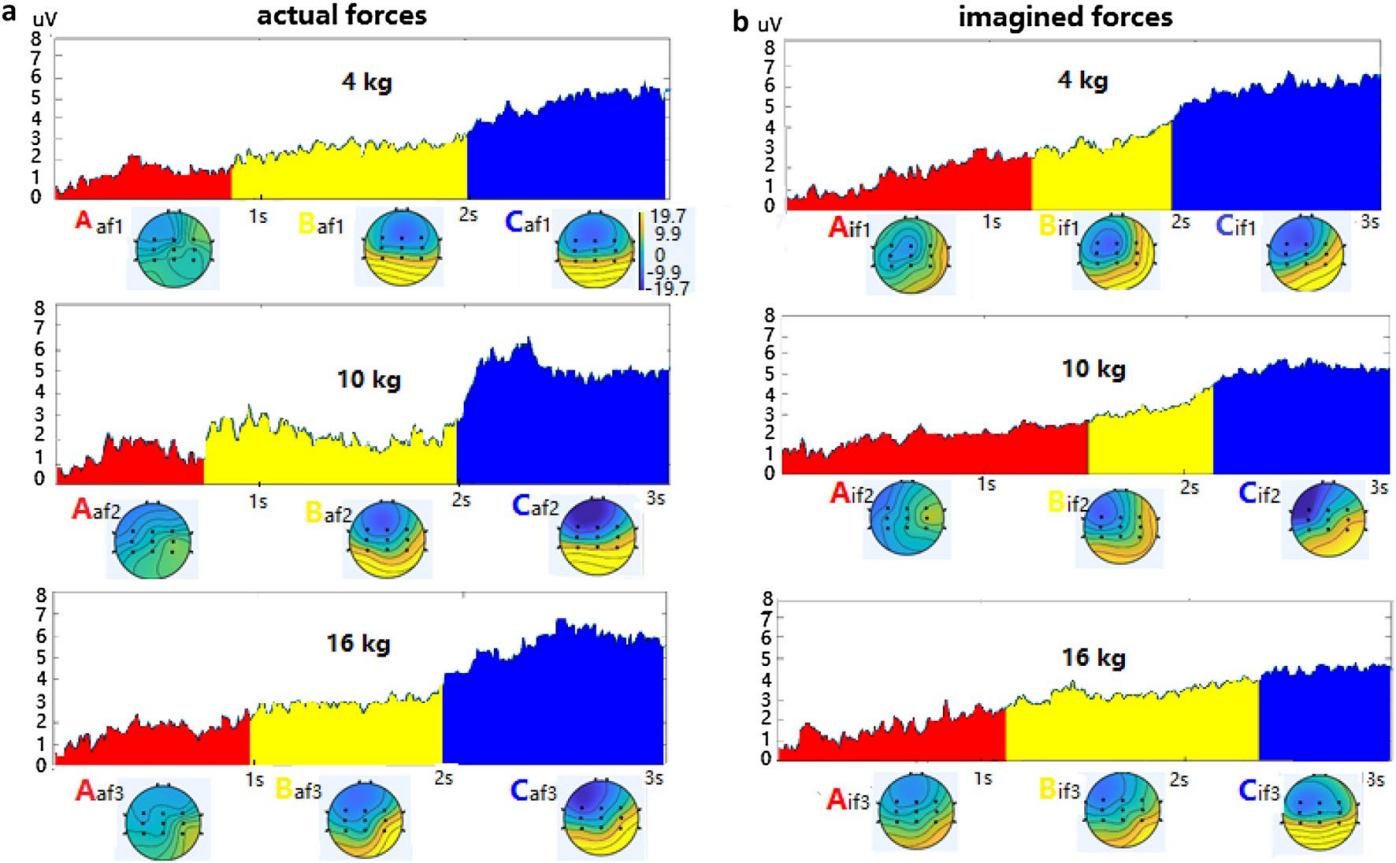
GFP curves and topographical maps of actual (**a**) and imagined (**b**) hand clenching forces of 4 kg, 10 kg and 16 kg. Three different dominant topographical maps represent the process of executed and imagined hand clenching force, labeled by A, B and C (A_af1_–C_af1_, A_af2_–C_af2_, A_af3_–C_af3_ for actual forces of 4 kg,10 kg, 16 kg in (**a**); A_if1_–C_if1_, A_if2_–C_if2_, A_if3_–C_if3_ for imagined forces of 4 kg,10 kg, 16 kg in (**b**)), and the GFP curves are segmented into three parts based on the topographic maps, expressed in red, yellow and blue. For topographic maps, the color map is the same for all plots. The yellow represents positive potential, and the blue represents negative potential

The GFPs of EEG signals were calculated according to formula (1), which represented the strength of the electric field over the brain at each instant. The GFP curves of actual and imagined hand clenching forces (each lasting 3 s) of 4 kg, 10 kg, and 16 kg for one subject are provided in Fig. 3. The local maximum values of GFP curve were obtained to generate topographic maps of the electrode array. These maps were submitted to the k-means clustering algorithm, which grouped these maps into a small set of clusters based on topographic similarity. The optimal number of clusters was determined by means of a cross-validation criterion (Koenig et al. 2014). For actual and imagined hand clenching force, the optimal number of clusters was all three, thus three representative topographical maps were obtained, labeled by A, B and C (i.e., A_af1_, B_af1_, C_af1_ for actual force of 4 kg; A_if1_, B_if1_, C_if1_ for imagined force of 4 kg), illustrated in Fig. 3. Finally, the original maps at maximum values of the GFP curves were assigned a label based on the map to which they best correlated. In Fig. 3, the GFP curves are segmented into three parts based on the topographic maps, expressed in red, yellow and blue. The transformation of topographical maps is A → B → C during the process of executed and imagined hand clenching forces. During the transition process, the left hemisphere is blue, but it gradually deepens; the right hemisphere is yellow, but it gradually deepens.

In Fig. 3a, the duration of maps C increases with the increase of the levels of the actual hand clenching forces, while the duration of map A of 10 kg is shortest and the duration of map B of 10 kg is longest. The amplitudes of map C also increase with the increase of the levels of the actual hand clenching forces. For each force, the amplitude of map A is smallest, that of map B is middle, and that of map C is biggest. For the imagined hand clenching forces in Fig. 3b, the variation of duration and amplitudes of maps A, B and C are different from that of actual forces, except the amplitudes of maps A, B and C for each force are increasing. Therefore, four parameters of maps A, B and C of the actual and imagined hand clenching forces were calculated for recognition, including duration, occurrence, time coverage and amplitude. The four parameters for recognition were calculated in a single trial, not in average epochs.

**Fig. 4:**
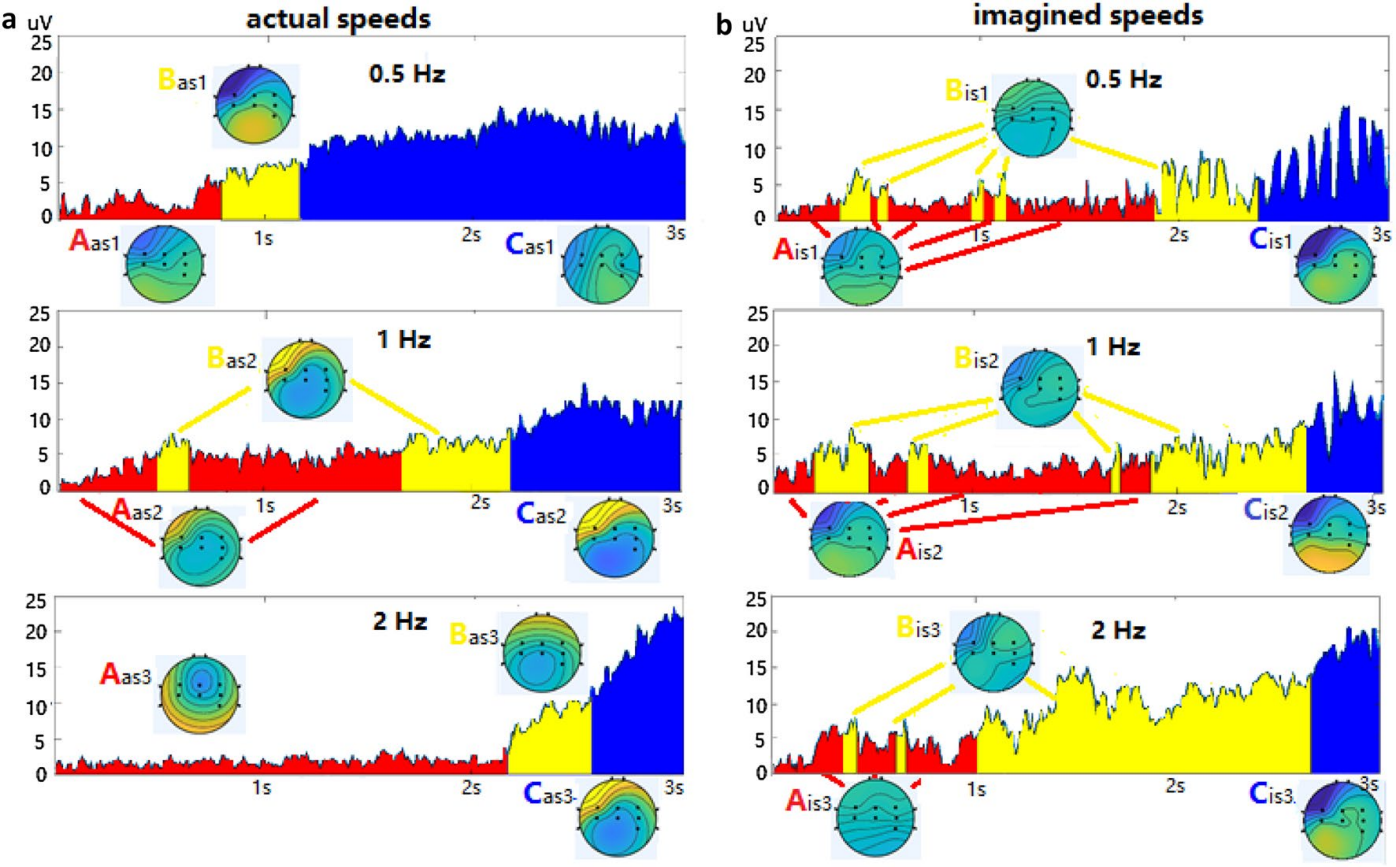
GFP curves and topographical maps of actual (**a**) and imagined (**b**) hand clenching speeds of 0.5 Hz, 1 Hz, and 2 Hz. Three different dominant topographical maps also represent the process of executed and imagined hand clenching speeds, labeled by A, B and C (A_as1_–C_as1_, A_as2_ – C_as2_, A_as3_ – C_as3_ for actual speeds of 0.5 Hz, 1 Hz, and 2 Hz in (**a**); A_is1_ – C_is1_, A_is2_ – C_is2_, A_is3_ – C_is3_ for imagined speeds of 0.5 Hz, 1 Hz, and 2 Hz in (**b**))

The procedures of extracting topographic maps for actual and imagined hand clenching speeds (lasting 3 s) of 0.5 Hz, 1 Hz, and 2 Hz were similar to that of forces. The GFP curves in actual and imagined hand clenching speed epochs are shown in Fig. 4, and the optimal number of clusters is also three, thus three different dominant topographical maps are obtained, labeled by A, B and C (i.e., A_as1_, B_as1_, C_as1_ for actual speed of 0.5 Hz; A_is1_, B_is1_, C_is1_ for imagined speed of 0.5 Hz). The GFP curves are also expressed in red, yellow and blue. However, the yellow of the three colors for speeds are scattered in the red in Fig. 4, are different from that of forces, in which the three colors appear sequentially and continuously in Fig. 3. For three topographic maps of actual speeds of 1 Hz and 2 Hz, the energy of left hemisphere of brain is gradually increased, while the energy of right hemisphere of brain is gradually decreased, which is opposite to that of forces. For three topographic maps of imagined speeds, the energy of left or right hemisphere doesn’t always increase or decrease. In Fig. 4a, the duration of maps A increases with the increase of the levels of the actual hand clenching speeds, while the duration of maps C decreases with the increase of the levels of the actual hand clenching speeds. Of the three maps for each speed, the duration of map B is shortest. The duration of maps A of 2 Hz is longest, and the amplitude of map A of 2 Hz is smallest. For the imagined hand clenching speeds in Fig. 4b, the variation of duration and amplitude of maps A, B and C are also different from that of actual speeds. Similarly, four parameters of maps A, B and C of actual and imagined hand clenching speeds in single trial were calculated for recognition.

### Brain Network Parameters of Actual and Imagined Forces and the Speeds of Hand Clenching

In the current study, the EEG networks of the Theta, Alpha and Beta bands were analyzed, and the clustering coefficient *C* and the shortest path length *L* of network attributes were calculated by BrainNetwork software (supported by Key Laboratory for Neuro Information of the Ministry of Education, School of Life Science and Technology, University of Electronic Science and Technology of China).

**Fig. 5:**
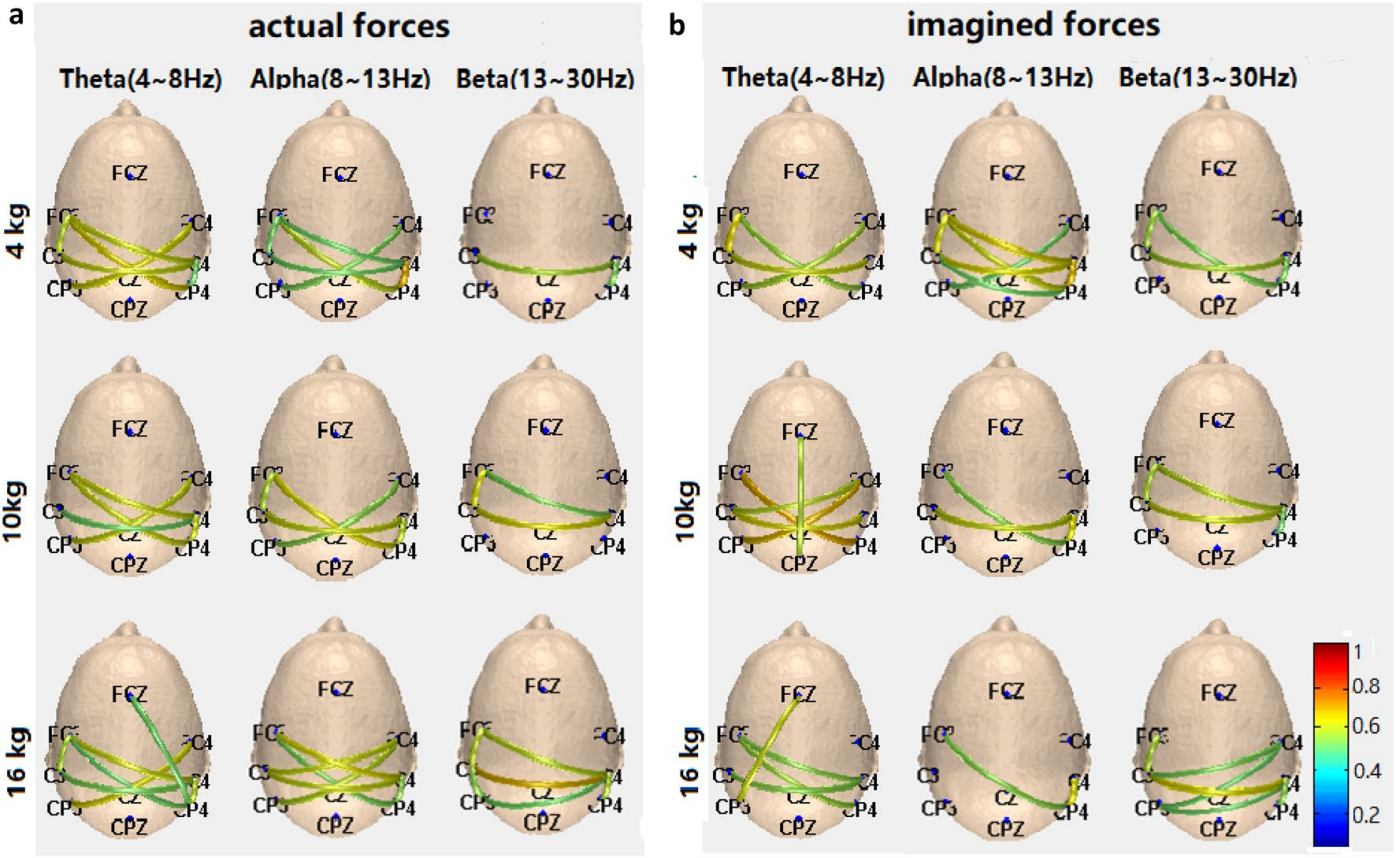
The network topologies of three bands of Theta, Alpha and Beta of the actual (**a**) and imagined (**b**) hand clenching forces

**Fig. 6:**
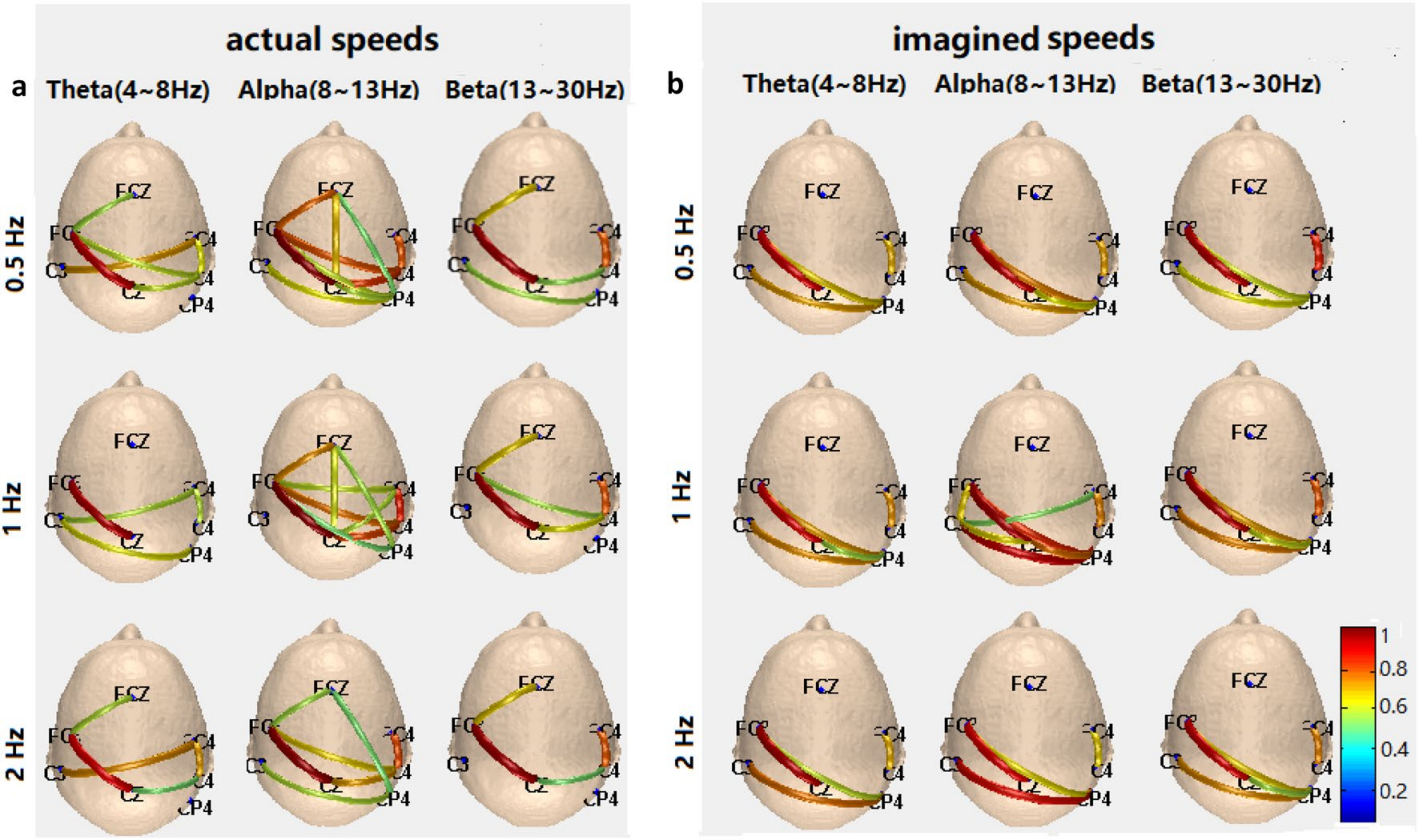
The network topologies of three bands of Theta, Alpha and Beta of the actual (**a**) and imagined (**b**) hand clenching speeds

The network topologies of three bands of Theta, Alpha and Beta of actual and imagined hand clenching forces and speeds are illustrated in Figs. 5 and 6, which show the strengths of connections of nine electrodes: the deeper the color is, the greater the strength is. For the force task, the connections of the Beta band increase with the increase of the levels of the actual hand clenching force in Fig. 5a, and the connections of the Beta band of the imagined hand clenching force have similar results in Fig. 5b. For the speed task, the connections of the Alpha and Beta bands decrease with the increase of the levels of the actual hand clenching speed in Fig. 6a, and the decrease of connections of the Alpha and Beta bands of the imagined hand clenching speed are not obvious, but the decrease of connections strength is obvious in Fig. 6b.

**Fig. 7:**
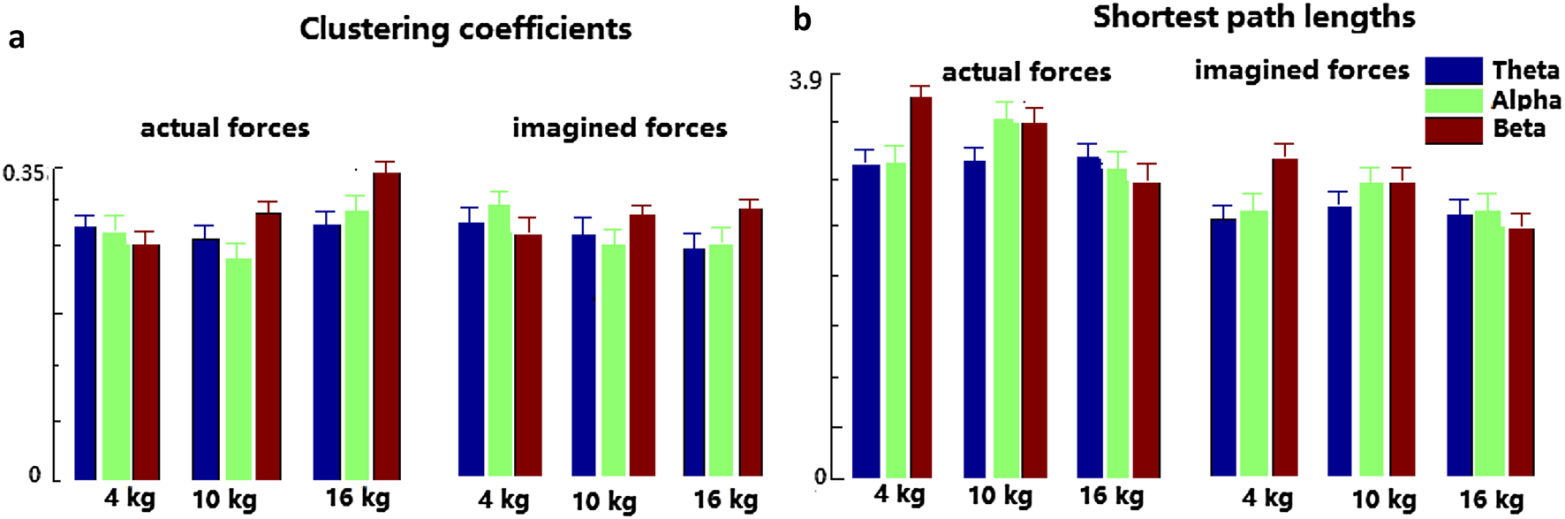
The clustering coefficients (**a**) and the shortest path lengths (**b**) of the actual and imagined hand clenching forces

The clustering coefficient and the shortest path length of the actual and imagined hand clenching force are illustrated in Fig. 7. For force task, the clustering coefficients of the Beta band (dark red bars) increase with the increase of the levels of the actual hand clenching force, whereas the shortest path length of the Beta band decreases with the increase of the levels of the actual hand clenching force, and the clustering coefficient as well as the shortest path length of the Beta band of the imagined hand clenching force have similar results. However, the clustering coefficient and the shortest path length of the Theta and Alpha bands are irregular.

**Fig. 8:**
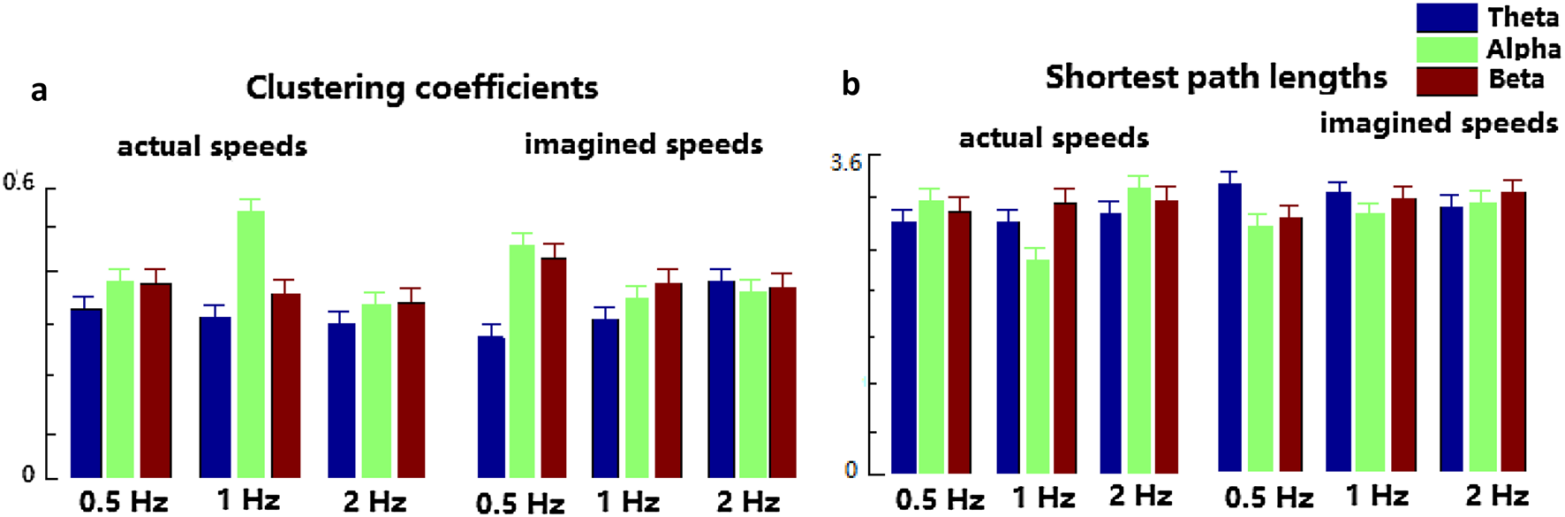
The clustering coefficients (**a**) and the shortest path length (**b**) of the actual and imagined hand clenching speeds

The clustering coefficient and the shortest path length of the actual and imagined hand clenching speeds are illustrated in Fig. 8. For the speed task, the clustering coefficients of the Beta band decrease with the increase of the levels of the actual hand clenching speed, while the shortest path length of the Beta band increases with the increase of the levels of the actual hand clenching speed, and the clustering coefficient and the shortest path length of the Beta band of the imagined hand clenching speed have similar results.

When the force and the speed tasks are compared, the clustering coefficient of forces (0.25–0.34) is smaller than that of speeds (0.26–0.52), and the shortest path length of forces (2.5–3.8) is larger than that of speeds (2.3–3.5) in Figs. 7 and 8.

### Classification

Based on the materials and methods proposed in this paper, after the investigation of actual and imagined hand clenching forces and speeds, five types of features were classified in three categories using LDA, SVM and ELMs. As mentioned previously, the number of five types of features was different, as presented in Table 1. The recognition results were obtained using leave-one-out cross-validation for three classification: 19 subjects for training (1710 trials concatenated) and 1 subject for testing (90 trials), repeated 20 times; every subject was used for testing for one time and the other 19 subjects for training, the final recognition rates were the average of those of 20 tests, presented in Tables 2 and 3.

**Table 2:** Recognition results of three levels of the actual and imagined hand clenching forces by single trial

	Actual hand clenching forces			Imagined hand clenching forces		
	LDA	ELM	SVM	LDA	ELM	SVM
Energy	0.48 ± 0.11	**0.95** ± **0.11**	**0.87** ± **0.14**	0.40 ± 0.11	**0.94** ± **0.12**	**0.94** ± **0.19**
Power spectrum of AR model	0.45 ± 0.11	**0.86** ± **0.17**	**0.96** ± **0.10**	0.46 ± 0.15	**0.89** ± **0.17**	**0.95** ± **0.12**
Wavelet packet coefficients	0.42 ± 0.11	**0.86** ± **0.20**	**0.83** ± **0.13**	0.36 ± 0.10	**0.91** ± **0.16**	**0.80** ± **0.16**
Topographical maps parameters	0.39 ± 0.13	**0.80** ± **0.10**	0.48 ± 0.15	0.42 ± 0.09	**0.77** ± **0.11**	0.46 ± 0.16
Brain network parameters	0.38 ± 0.08	0.52 ± 0.22	0.57 ± 0.19	0.37 ± 0.08	0.52 ± 0.17	0.56 ± 0.20
Combined features (three traditional features)	0.50 ± 0.13	**0.94** ± **0.10**	**0.95** ± **0.13**	0.57 ± 0.11	**0.93** ± **0.12**	**0.92** ± **0.14**
Combined features (all five features)	0.49 ± 0.11	**0.96** ± **0.07**	**0.97** ± **0.08**	0.58 ± 0.15	**0.96** ± **0.05**	**0.95** ± **0.07**

Bold values indicate that the recognition rates are better than the others

**Table 3:** Recognition results of three levels of actual and imagined hand clenching speeds by single trial

	Actual hand clenching speeds			Imagined hand clenching speeds		
	LDA	ELM	SVM	LDA	ELM	SVM
Energy	0.40 ± 0.08	0.66 ± 0.04	0.65 ± 0.03	0.42 ± 0.05	0.66 ± 0.03	0.65 ± 0.05
Power spectrum of AR model	0.63 ± 0.04	0.64 ± 0.04	0.63 ± 0.03	0.66 ± 0.03	0.66 ± 0.03	0.65 ± 0.05
Wavelet packet coefficients	0.59 ± 0.09	0.67 ± 0.05	0.66 ± 0.10	0.58 ± 0.11	0.65 ± 0.03	0.66 ± 0.09
Topographical maps parameters	0.42 ± 0.07	0.63 ± 0.07	0.64 ± 0.08	0.47 ± 0.06	0.64 ± 0.12	0.65 ± 0.05
Brain network parameters	0.83 ± 0.12	**0.97** ± **0.12**	**0.97** ± **0.15**	0.72 ± 0.02	**1.00** ± **0.00**	**1.00** ± **0.00**
Combined features (three traditional features)	0.56 ± 0.09	0.65 ± 0.05	0.66 ± 0.03	0.55 ± 0.12	0.66 ± 0.03	0.66 ± 0.06
Combined features (all five features)	0.84 ± 0.05	**0.98** ± **0.03**	**0.98** ± **0.06**	0.79 ± 0.09	**1.00** ± **0.00**	**1.00** ± **0.00**

Bold values indicate that the recognition rates are better than the others

Because of the difference of the EEG signals between subjects, it is necessary to train specific classifiers for the specific subjects to manipulate BCIs. We also built a classifier for each subject, two sessions for training and one session for testing. The recognition results were shown in Supplementary Tables 4–7, which were attached as additional materials.

Not only the EEG signals between the different subjects are different, but also the EEG signals of the same subject at different times are also different. Therefore, for topographical maps features employed for recognition, it needs a method to match the topographical maps under the different conditions. We calculated the correlation coefficients between the maps of each trial of each subject and the typical topographical maps previously extracted under different conditions (i.e., A_af1_, B_af1_, C_af1_, A_af2_, B_af2_, C_af2,_ A_af3_, B_af3_, C_af3_ for the typical topographical maps A, B and C of actual hand clenching at 4 kg, 10 kg, and 16 kg in Fig. 3). If the correlation coefficient of the maps was greater than that of a certain value (0.55 for forces and 0.45 for speeds), it was considered that the two maps were corresponding in time and matched. Then, the matching maps parameters were calculated for classification. The average correlation coefficients between the maps of all trials of all subjects and the typical topographical maps were calculated, shown in Supplementary Tables 8–11, and the permutation test for all five types features were used to determine chance levels of the LDA, ELM and SVM, shown in Supplementary Table 12, which were all attached as additional materials.

In Table 2, the classification results of LDA were poor, with a recognition rate of 37%–57%, but that of ELMs and SVM were good, with the recognition rate of 46%–97%. In Table 2, the recognition rates of energy, power spectrum of the AR model and wavelet packet coefficients were higher, above 80% using ELMs and SVM, slightly worse for topographical maps parameters, and worst for brain network parameters. For topographical maps parameters, the recognition rates of the actual and imagined hand clenching forces were 80% and 77% using ELMs, 48% and 46% using SVM. For brain network parameters, the recognition rates of the actual and imagined hand clenching forces were 52% and 52% using ELMs, 57% and 56% using SVM, which were far lower than the other four types of features. The recognition results of combination of three traditional types of features were not remarkably improved, but the recognition rates of five types of combined features were higher than that of each type of feature: 96% and 96% using ELMs, 97% and 95% using SVM.

It could also be seen from Table 3 that the classification results of LDA were poor, and ELMs and SVM were better. Among the five types of features, the recognition rates of energy, power spectrum of the AR model and wavelet packet coefficients and topographical maps parameters were low, only 40%–66%. By contrast, the classification results of brain network parameters were very good, with recognition rates of 83% and 72% for LDA, 97% and 100% for ELMs, as well as 97% and 100% for SVM. The recognition results of combination of three traditional types of features were also not remarkably improved, similarly to those for energy, the power spectrum of AR, wavelet packet coefficient and topographical maps parameters. While the recognition rates of the five combined features were higher than that of each type of feature: 84% and 79% using LDA, 98% and 100% using ELM, as well as 98% and 100% using SVM.

## Discussion

### Topographical Maps Parameters for Identifying Hand Clenching Forces and Speeds

In the current study, the EEG signals of the actual/imagined hand clenching forces/speeds, analyzed by microstates, were expressed as the time series of different brain topologies. As illustrated in Figs. 3 and 4, the EEG signals of the actual/imagined hand clenching forces/speeds were composed of topographical maps A, B and C. Four parameters of maps A, B and C of the actual/imagined hand clenching forces/speeds were calculated for recognition. And the recognition results showed that topographical maps parameters were better for identifying the actual/imagined hand clenching forces than for speeds in Tables 2 and 3.

In fact, the parameters of microstate and sequence are not random, but follow certain rules. The arrangement of the microstate order is called syntax. For example, for schizophrenic patients, it was found that the duration of the two typical microstates became shorter, and the sequence of the four microstates (syntax) became chaotic (Kikuchi et al. 2011). For healthy subjects, the duration of the microstates depended on the changes in the arousal sleep cycle, which became shorter in deep sleep and grew longer in meditation. In addition to duration in the current study, occurrence, time coverage and amplitude of topographical maps A, B, C of actual/imagined hand clenching forces/speeds were also adopted, which reflected the characteristics of neural activity under different tasks from different aspects. Thus, with the different levels of actual/imagined hand clenching forces/speeds, the characteristics of neural activity were different, and the parameters of the microstates were also different, which was the fundamental reason why the topographical maps parameters could be used to identify the hand clenching force/speed.

However, the recognition results of the topographical maps parameters for hand clenching forces and speeds were different, good for forces, poor for speeds. In Fig. 3, for actual and imagined hand clenching forces, the energy of left hemispheres of topographic maps A, B, C are gradually decreased (blue) and the energy of right hemispheres are gradually increased (yellow). While for actual and imagined hand clenching speeds in Fig. 4, the energy of left hemisphere of three topographic maps of actual speeds of 1 Hz and 2 Hz is gradually increased, and the energy of right hemisphere is gradually decreased, which is opposite to that of forces. And the energy of left or right hemisphere of imagined speeds doesn’t always increase or decrease. This indicated that the change of neural activity under force tasks showed certain regularity, but the change of neural activity under speed tasks was not. In addition, as can be seen from Supplementary Tables 8–11 in additional materials, the average correlation coefficients between the maps of each trial and the typical topographic maps of forces were greater than that of speeds. This might mean that the variations of force maps of each trial were smaller than that of speed, and were closer to the typical topographic maps. These might be one of the reasons why the topographical maps parameters were better for identifying the actual/imagined hand clenching forces than for identifying speeds.

### Brain Network Parameters for Identifying the Hand Clenching Forces and Speeds

The network topology plays crucial role in the function of the brain network and the dynamics of the whole system, which influences the propagation of neural signals. Average path length and the clustering coefficient are often employed to characterize the topological and dynamic properties of networks. The clustering coefficient measures the degree of collectivization of a network. The higher the clustering coefficient is, the higher the degree of collectivization on behalf of the whole brain network is, and the higher the efficiency of the corresponding network is. The shortest path length reflects the dispersion and connectivity of the network structure, the shorter the shortest path is, the more compact the network structure is, and the better the connectivity of the network is. Zhou demonstrated that a shorter reaction time was correlated with a shorter path length in the gamma band using resting-sate EEG (Zhou et al. 2012). Douw found that an increased clustering coefficient in delta, theta and gamma bands was correlated to better cognition using resting-state MEG (Douw et al. 2011).

In the current study, for brain network analysis of the actual/imagined hand clenching force/speed, the clustering coefficient and the shortest path length of the Theta, Alpha and Beta bands were calculated, as illustrated in Figs. 7 and 8. For the force task, the clustering coefficient of the Beta band increase with the increase of the levels of actual hand clenching force, whereas the shortest path length of the Beta band decrease with the increase of the levels of the actual hand clenching force. This indicated that the higher the actual force of hand clenching in Beta band was, the higher the efficiency of the corresponding brain network was, and the better the connectivity of the brain network was. The imagined forces of hand clenching were similar to executed forces on physiological basis, which had similar results. These results were well expressed in Fig. 5. This indicated that the levels of actual/imagined hand clenching forces were positively correlated to the connection of Beta band.

For the speed task, the clustering coefficient of the Beta band decrease with the increase of the levels of the actual hand clenching speed, while the shortest path length of the Beta band increase with the increase of the levels of the actual hand clenching speed, which was the opposite of that of the force task. This indicated that the higher the actual speed of hand clenching was, in Beta band, the lower the efficiency of the corresponding brain network was, and the worse the connectivity of the brain network was. These results were also consistent with those in Fig. 6. This demonstrated that the levels of actual/imagined hand clenching speeds were negatively correlated to the connection of Beta band.

When the force task was compared to the speed task, the clustering coefficient of the force was smaller than that of the speed, and the shortest path length of the force was larger than that of the speed. This meant that the brain network of force was less efficient and had less connectivity than that of speed. It could be demonstrated that the communication between any two nodes of brain network at speed tasks could be completed quickly, especially for the FC3 and Cz nodes, of which the line was most red in Fig. 6.

For recognition of the actual/imagined hand clenching forces in Table 2, the best recognition rate was 57%, and the brain network parameters of the five types of features were the worst. By contrast, the recognition results of three levels of actual/imagined hand clenching speeds were very good, with a recognition rate of 83%–100% in Table 3. The difference of recognition rates for force and speed task suggested that a more efficient brain network may facilitate the recognition of force/speed of hand clenching, whether executed or imagined.

### Combined Features for Identifying the Hand Clenching Forces and Speeds

In Tables 2 and 3, the recognition ability of energy, the power spectrum of the AR model, wavelet packet coefficients and topographical maps parameters for identifying the hand clenching forces were better, worse for speeds. On the contrary, the recognition ability of brain network parameters for forces were worse, but better for speeds; therefore, the recognition rate might be further improved by combining these five types of features. As we know that increasing the number of features might increase accuracy asymptotically, we compared the recognition results of the combination of three traditional types of features and all five types of features. The results illustrated that the recognition results of the combination of three traditional types of features were not remarkably improved; these results were similar to those of energy and the power spectrum of the AR model but better than those of wavelet packet coefficients. By contrast, the recognition results of the combination of all five types of features were remarkably improved, especially for LDA. The recognition rate of ELM and SVM were as high as 95%–97% for forces, 98%–100% for speeds. This suggested that the characteristics of the topography and brain network information were beneficial to the improvement of the recognition results.

## Conclusion

In the practical brain control robot system, it is necessary to provide the robot with additional force and speed control instructions, and it is also necessary to find new features to improve the classification accuracy. In the current study, topographical maps parameters and brain network parameters as the new classification features were calculated and combined with the traditional features (energy, power spectrum of the AR model and wavelet packet coefficients) to further improve the classification accuracy of a single trial.

The results of single-trial recognition of the actual/imagined forces/speeds of hand clenching based on LDA, ELMs and SVM as classifiers indicated that topographical maps parameters were better for identifying the hand clenching force, and the recognition results of brain network parameters were better for identifying hand clenching speed. The combination of five types of features further improved the recognition rates, with a recognition rate of 97% for the hand clenching force and 100% for hand clenching speed. This indicated that topographical maps and brain network parameters could be used as new characteristics for decoding the actual/imagined forces and speeds of hand clenching. Combined with traditional characteristics, the combination of five types of characteristics could significantly improve the recognition rate of actual/ force and speed of hand clenching. Future research work needs to validate online classification performance of these parameters.

## Electronic supplementary material

Below is the link to the electronic supplementary material.

Supplementary material 1 (DOCX 48 KB)

Supplementary material 2 (DOCX 27 KB)
