# Supplementary material for: Single-Trial Recognition of Imagined Forces and Speeds of Hand Clenching Based on Brain Topography and Brain Network
Source: Brain Topogr. 2018 Dec 31;32(2):240–54. doi: 10.1007/s10548-018-00696-3 (PMC6373301; doi:10.1007/s10548-018-00696-3)
Supplement: Supplementary file 1 — Supplementary material 1 (DOCX 48 KB) [file 10548_2018_696_MOESM1_ESM.docx]

The recognition results of three levels of the actual/imagined hand clenching forces/speeds by single trial for individual subject were shown in Tables 4~7, in which Energy is abbreviated as E, Power Spectrum of AR Model is abbreviated as PS, Wavelet Packet Coefficients is abbreviated as WP, Topographical maps Parameters is abbreviated as TP, Brain Network Parameters is abbreviated as BN, Combined Features (three traditional features) is abbreviated as CF3, and Combined Features (all five features) is abbreviated as CF5.

Table 4 Recognition results of three levels of the actual hand clenching forces by single trial for individual subject.

|  | Subject1 | | | Subject2 | | | Subject3 | | | | Subject4 | | | |
| --- | --- | --- | --- | --- | --- | --- | --- | --- | --- | --- | --- | --- | --- | --- |
|  | LDA | ELM | SVM | LDA | ELM | SVM | LDA | ELM | SVM | | LDA | ELM | SVM | |
| E | 0.20 | 0.57 | 0.63 | 0.40 | 0.54 | 0.63 | 0.33 | 0.47 | 0.47 | | 0.53 | 0.27 | 0.40 | |
| PS | 0.47 | 0.53 | 0.33 | 0.33 | 0.63 | 0.63 | 0.13 | 0.53 | 0.62 | | 0.33 | 0.40 | 0.40 | |
| WP | 0.33 | 0.60 | 0.47 | 0.33 | 0.63 | 0.63 | 0.33 | 0.33 | 0.27 | | 0.40 | 0.40 | 0.27 | |
| TP | 0.33 | 0.60 | 0.47 | 0.20 | 0.63 | 0.57 | 0.33 | 0.53 | 0.40 | | 0.33 | 0.47 | 0.47 | |
| BN | 0.60 | 0.33 | 0.33 | 0.33 | 0.63 | 0.64 | 0.47 | 0.53 | 0.33 | | 0.40 | 0.13 | 0.40 | |
| CF3 | 0.33 | 0.57 | 0.63 | 0.47 | 0.53 | 0.64 | 0.27 | 0.54 | 0.47 | | 0.47 | 0.57 | 0.57 | |
| CF5 | 0.33 | 0.63 | 0.60 | 0.33 | 0.64 | 0.63 | 0.40 | 0.63 | 0.53 | | 0.40 | 0.67 | 0.53 | |
|  | Subject5 | | | Subject6 | | | Subject7 | | | | Subject8 | | | |
|  | LDA | ELM | SVM | LDA | ELM | SVM | LDA | ELM | | SVM | LDA | ELM | | SVM |
| E | 0.33 | 0.77 | 0.80 | 0.53 | 0.97 | 0.87 | 0.13 | 0.62 | | 0.75 | 0.33 | 0.67 | | 0.72 |
| PS | 0.53 | 0.82 | 0.83 | 0.40 | 0.78 | 0.85 | 0.13 | 0.93 | | 0.92 | 0.33 | 0.76 | | 0.72 |
| WP | 0.77 | 0.75 | 0.78 | 0.40 | 0.76 | 0.89 | 0.73 | 0.92 | | 0.92 | 0.33 | 0.78 | | 0.83 |
| TP | 0.60 | 0.74 | 0.83 | 0.53 | 0.93 | 0.74 | 0.40 | 0.83 | | 0.87 | 0.40 | 0.73 | | 0.73 |
| BN | 0.33 | 0.27 | 0.33 | 0.33 | 0.33 | 0.33 | 0.33 | 0.33 | | 0.33 | 0.60 | 0.92 | | 0.93 |
| CF3 | 0.43 | 0.75 | 0.87 | 0.60 | 0.97 | 0.98 | 0.20 | 0.92 | | 0.92 | 0.47 | 0.87 | | 0.91 |
| CF5 | 0.57 | 0.84 | 0.88 | 0.60 | 1.00 | 1.00 | 0.20 | 0.92 | | 0.93 | 0.53 | 0.92 | | 0.93 |
|  | Subject9 | | | Subject10 | | | Subject11 | | | | Subject12 | | | |
|  | LDA | ELM | SVM | LDA | ELM | SVM | LDA | ELM | | SVM | LDA | ELM | | SVM |
| E | 0.61 | 0.65 | 0.66 | 0.13 | 0.8 | 0.73 | 0.13 | 0.88 | | 0.94 | 0.33 | 0.73 | | 0.53 |
| PS | 0.64 | 0.66 | 0.66 | 0.27 | 0.73 | 0.47 | 0.60 | 0.78 | | 0.82 | 0.33 | 0.47 | | 0.60 |
| WP | 0.62 | 0.64 | 0.63 | 0.53 | 0.47 | 0.33 | 0.80 | 0.89 | | 0.89 | 0.33 | 0.67 | | 0.40 |
| TP | 0.60 | 0.55 | 0.53 | 0.67 | 0.47 | 0.53 | 0.53 | 0.87 | | 0.93 | 0.40 | 0.27 | | 0.40 |
| BN | 0.33 | 0.33 | 0.33 | 0.33 | 0.28 | 0.28 | 0.33 | 0.33 | | 0.33 | 0.27 | 0.47 | | 0.60 |
| CF3 | 0.60 | 0.55 | 0.66 | 0.40 | 0.87 | 0.53 | 0.53 | 0.88 | | 0.89 | 0.53 | 0.87 | | 0.40 |
| CF5 | 0.64 | 0.65 | 0.68 | 0.20 | 0.87 | 0.53 | 0.6 | 0.88 | | 0.82 | 0.33 | 0.87 | | 0.53 |
|  | Subject13 | | | Subject14 | | | Subject15 | | | | Subject16 | | | |
|  | LDA | ELM | SVM | LDA | ELM | SVM | LDA | ELM | | SVM | LDA | ELM | | SVM |
| E | 0.40 | 0.89 | 0.73 | 0.33 | 0.83 | 0.88 | 0.85 | 0.86 | | 0.96 | 0.53 | 0.92 | | 0.89 |
| PS | 0.27 | 0.90 | 0.93 | 0.20 | 0.86 | 0.87 | 0.33 | 0.86 | | 0.97 | 0.20 | 0.96 | | 0.97 |
| WP | 0.40 | 0.87 | 0.87 | 0.89 | 0.88 | 0.88 | 0.90 | 0.87 | | 0.96 | 0.87 | 0.94 | | 0.95 |
| TP | 0.47 | 0.27 | 0.67 | 0.53 | 0.87 | 0.87 | 0.67 | 0.83 | | 0.95 | 0.67 | 0.93 | | 0.99 |
| BN | 0.47 | 0.40 | 0.40 | 0.67 | 0.87 | 0.80 | 0.60 | 0.88 | | 0.98 | 0.73 | 0.95 | | 0.96 |
| CF3 | 0.27 | 0.92 | 0.93 | 0.53 | 0.88 | 0.88 | 0.83 | 0.80 | | 0.98 | 0.67 | 0.93 | | 0.95 |
| CF5 | 0.40 | 0.94 | 0.93 | 0.53 | 0.90 | 0.90 | 0.83 | 0.86 | | 1.00 | 0.67 | 0.93 | | 0.95 |
|  | Subject17 | | | Subject18 | | | Subject19 | | | | Subject20 | | | |
|  | LDA | ELM | SVM | LDA | ELM | SVM | LDA | ELM | | SVM | LDA | ELM | | SVM |
| E | 0.33 | 0.77 | 0.67 | 0.33 | 0.67 | 0.82 | 0.33 | 0.77 | | 0.69 | 0.33 | 0.87 | | 0.77 |
| PS | 0.33 | 0.75 | 0.69 | 0.88 | 0.95 | 0.94 | 0.33 | 0.68 | | 0.69 | 0.33 | 0.75 | | 0.69 |
| WP | 0.86 | 0.92 | 0.89 | 0.92 | 0.94 | 0.94 | 0.67 | 0.83 | | 0.93 | 0.75 | 0.82 | | 0.79 |
| TP | 0.67 | 0.78 | 0.82 | 0.53 | 0.97 | 0.93 | 0.53 | 0.73 | | 0.87 | 0.67 | 0.78 | | 0.82 |
| BN | 0.33 | 0.33 | 0.33 | 0.80 | 0.98 | 0.93 | 0.6 | 0.93 | | 0.73 | 0.33 | 0.33 | | 0.33 |
| CF3 | 0.83 | 0.92 | 0.95 | 0.53 | 1.00 | 0.94 | 0.73 | 0.93 | | 0.93 | 0.83 | 0.82 | | 0.85 |
| CF5 | 0.83 | 0.92 | 0.92 | 0.67 | 1.00 | 0.94 | 0.33 | 0.93 | | 0.93 | 0.83 | 0.87 | | 0.89 |

Table 5 Recognition results of three levels of the imagined hand clenching forces by single trial for individual subject.

|  | Subject1 | | | Subject2 | | | Subject3 | | | Subject4 | | | | |
| --- | --- | --- | --- | --- | --- | --- | --- | --- | --- | --- | --- | --- | --- | --- |
|  | LDA | ELM | SVM | LDA | ELM | SVM | LDA | ELM | SVM | LDA | ELM | | | SVM |
| E | 0.40 | 0.60 | 0.40 | 0.53 | 0.33 | 0.40 | 0.47 | 0.13 | 0.27 | 0.27 | 0.27 | | | 0.20 |
| PS | 0.20 | 0.67 | 0.67 | 0.40 | 0.53 | 0.33 | 0.47 | 0.33 | 0.40 | 0.40 | 0.33 | | | 0.40 |
| WP | 0.53 | 0.40 | 0.33 | 0.33 | 0.33 | 0.40 | 0.27 | 0.60 | 0.40 | 0.33 | 0.53 | | | 0.67 |
| TP | 0.40 | 0.53 | 0.60 | 0.27 | 0.33 | 0.27 | 0.27 | 0.53 | 0.40 | 0.27 | 0.27 | | | 0.40 |
| BN | 0.53 | 0.40 | 0.33 | 0.33 | 0.33 | 0.27 | 0.27 | 0.33 | 0.27 | 0.40 | 0.13 | | | 0.20 |
| CF3 | 0.40 | 0.60 | 0.67 | 0.27 | 0.27 | 0.33 | 0.47 | 0.27 | 0.27 | 0.20 | 0.20 | | | 0.33 |
| CF5 | 0.47 | 0.73 | 0.67 | 0.40 | 0.40 | 0.33 | 0.47 | 0.53 | 0.40 | 0.47 | 0.27 | | | 0.40 |
|  | Subject5 | | | Subject6 | | | Subject7 | | | Subject8 | | | | |
|  | LDA | ELM | SVM | LDA | ELM | SVM | LDA | ELM | SVM | LDA | ELM | | | SVM |
| E | 0.33 | 0.67 | 0.72 | 0.33 | 0.74 | 0.72 | 0.33 | 0.75 | 0.74 | 0.13 | 0.76 | | | 0.69 |
| PS | 0.33 | 0.69 | 0.75 | 0.47 | 0.72 | 0.76 | 0.53 | 0.85 | 0.81 | 0.13 | 0.72 | | | 0.66 |
| WP | 0.85 | 0.88 | 0.88 | 0.47 | 0.84 | 0.76 | 0.80 | 0.82 | 0.81 | 0.83 | 0.88 | | | 0.78 |
| TP | 0.73 | 0.82 | 0.82 | 0.53 | 0.93 | 0.89 | 0.53 | 0.77 | 0.73 | 0.60 | 0.93 | | | 0.93 |
| BN | 0.33 | 0.33 | 0.33 | 0.33 | 0.33 | 0.33 | 0.33 | 0.33 | 0.33 | 0.67 | 0.77 | | | 0.75 |
| CF3 | 0.33 | 0.88 | 0.88 | 0.33 | 0.93 | 0.76 | 0.53 | 0.78 | 0.81 | 0.13 | 0.88 | | | 0.75 |
| CF5 | 0.47 | 0.92 | 0.92 | 0.33 | 0.93 | 0.95 | 0.33 | 0.85 | 0.87 | 0.47 | 0.93 | | | 0.93 |
|  | Subject9 | | | Subject10 | | | Subject11 | | | Subject12 | | | | |
|  | LDA | ELM | SVM | LDA | ELM | SVM | LDA | ELM | SVM | LDA | ELM | | | SVM |
| E | 0.2 | 0.77 | 0.69 | 0.60 | 0.60 | 0.60 | 0.33 | 0.66 | 0.66 | 0.27 | 0.93 | | | 0.67 |
| PS | 0.2 | 0.81 | 0.73 | 0.40 | 0.73 | 0.47 | 0.33 | 0.87 | 0.77 | 0.60 | 0.80 | | | 0.53 |
| WP | 0.77 | 0.89 | 0.85 | 0.53 | 0.33 | 0.40 | 0.81 | 0.87 | 0.87 | 0.40 | 0.60 | | | 0.40 |
| TP | 0.60 | 0.89 | 0.86 | 0.57 | 0.47 | 0.47 | 0.6 | 0.92 | 0.93 | 0.60 | 0.20 | | | 0.47 |
| BN | 0.33 | 0.33 | 0.33 | 0.33 | 0.47 | 0.33 | 0.33 | 0.33 | 0.33 | 0.47 | 0.53 | | | 0.67 |
| CF3 | 0.33 | 0.92 | 0.94 | 0.33 | 0.53 | 0.67 | 0.67 | 0.92 | 0.93 | 0.33 | 0.93 | | | 0.33 |
| CF5 | 0.47 | 0.92 | 0.96 | 0.47 | 0.73 | 0.47 | 0.27 | 0.95 | 0.95 | 0.4 | 0.95 | | | 0.87 |
|  | Subject13 | | | Subject14 | | | Subject15 | | | Subject16 | | | | |
|  | LDA | ELM | SVM | LDA | ELM | SVM | LDA | ELM | SVM | LDA | ELM | | | SVM |
| E | 0.20 | 0.93 | 0.93 | 0.80 | 0.85 | 0.87 | 0.40 | 0.78 | 0.68 | 0.40 | 0.88 | | | 0.92 |
| PS | 0.53 | 0.85 | 0.78 | 0.33 | 0.78 | 0.75 | 0.47 | 0.67 | 0.78 | 0.40 | 0.77 | | | 0.65 |
| WP | 0.67 | 0.87 | 0.80 | 0.85 | 0.89 | 0.94 | 0.85 | 0.88 | 0.68 | 0.82 | 0.67 | | | 0.72 |
| TP | 0.60 | 0.60 | 0.60 | 0.47 | 0.78 | 0.68 | 0.60 | 0.87 | 0.80 | 0.60 | 0.78 | | | 0.93 |
| BN | 0.47 | 0.73 | 0.47 | 0.6 | 0.76 | 0.93 | 0.93 | 0.93 | 0.93 | 0.67 | 0.88 | | | 0.93 |
| CF3 | 0.13 | 1.00 | 0.87 | 0.22 | 0.93 | 0.94 | 0.60 | 0.95 | 0.88 | 0.53 | 0.88 | | | 0.93 |
| CF5 | 0.53 | 1.00 | 0.87 | 0.22 | 0.93 | 0.96 | 0.91 | 0.98 | 1.00 | 0.40 | 0.88 | | | 0.93 |
|  | Subject17 | | | Subject18 | | | Subject19 | | | Subject20 | | | | |
|  | LDA | ELM | SVM | LDA | ELM | SVM | LDA | ELM | SVM | LDA | | ELM | SVM | |
| E | 0.40 | 0.55 | 0.58 | 0.20 | 0.78 | 0.72 | 0.47 | 0.67 | 0.76 | 0.33 | | 0.57 | 0.58 | |
| PS | 0.40 | 0.43 | 0.47 | 0.40 | 0.64 | 0.57 | 0.33 | 0.56 | 0.63 | 0.57 | | 0.67 | 0.58 | |
| WP | 0.33 | 0.67 | 0.78 | 0.47 | 0.78 | 0.72 | 0.80 | 0.76 | 0.87 | 0.55 | | 0.58 | 0.68 | |
| TP | 0.60 | 0.76 | 0.65 | 0.47 | 0.55 | 0.47 | 0.47 | 0.67 | 0.80 | 0.60 | | 0.67 | 0.60 | |
| BN | 0.33 | 0.33 | 0.33 | 0.73 | 0.80 | 0.82 | 0.53 | 0.73 | 0.67 | 0.53 | | 0.73 | 0.73 | |
| CF3 | 0.33 | 0.78 | 0.80 | 0.33 | 0.86 | 0.82 | 0.27 | 0.80 | 0.80 | 0.60 | | 0.75 | 0.78 | |
| CF5 | 0.78 | 0.80 | 0.84 | 0.33 | 0.88 | 0.85 | 0.27 | 0.82 | 0.89 | 0.71 | | 0.88 | 0.80 | |

Table 6 Recognition results of three levels of the actual hand clenching speeds by single trial for individual subject.

|  | Subject1 | | | Subject2 | | | Subject3 | | | Subject4 | | | |
| --- | --- | --- | --- | --- | --- | --- | --- | --- | --- | --- | --- | --- | --- |
|  | LDA | ELM | SVM | LDA | ELM | SVM | LDA | ELM | SVM | LDA | ELM | | SVM |
| E | 0.23 | 0.45 | 0.47 | 0.56 | 0.22 | 0.44 | 0.34 | 0.47 | 0.42 | 0.33 | 0.44 | | 0.44 |
| PS | 0.22 | 0.11 | 0.35 | 0.22 | 0.22 | 0.33 | 0.33 | 0.33 | 0.33 | 0.56 | 0.22 | | 0.33 |
| WP | 0.43 | 0.33 | 0.43 | 0.44 | 0.33 | 0.33 | 0.44 | 0.36 | 0.34 | 0.44 | 0.12 | | 0.33 |
| TP | 0.22 | 0.22 | 0.22 | 0.33 | 0.22 | 0.11 | 0.33 | 0.11 | 0.11 | 0.33 | 0.23 | | 0.26 |
| BN | 0.64 | 0.68 | 0.68 | 0.56 | 0.64 | 0.56 | 0.47 | 0.45 | 0.47 | 0.44 | 0.60 | | 0.64 |
| CF3 | 0.43 | 0.22 | 0.43 | 0.33 | 0.56 | 0.22 | 0.33 | 0.44 | 0.33 | 0.33 | 0.33 | | 0.44 |
| CF5 | 0.43 | 0.72 | 0.72 | 0.33 | 0.67 | 0.63 | 0.33 | 0.54 | 0.53 | 0.22 | 0.67 | | 0.73 |
|  | Subject5 | | | Subject6 | | | Subject7 | | | Subject8 | | | |
|  | LDA | ELM | SVM | LDA | ELM | SVM | LDA | ELM | SVM | LDA | ELM | | SVM |
| E | 0.24 | 0.33 | 0.47 | 0.67 | 0.67 | 0.44 | 0.24 | 0.44 | 0.44 | 0.22 | 0.44 | | 0.44 |
| PS | 0.47 | 0.34 | 0.34 | 0.22 | 0.22 | 0.33 | 0.33 | 0.33 | 0.33 | 0.22 | 0.44 | | 0.33 |
| WP | 0.47 | 0.24 | 0.33 | 0.44 | 0.56 | 0.33 | 0.44 | 0.33 | 0.33 | 0.44 | 0.33 | | 0.33 |
| TP | 0.33 | 0.33 | 0.33 | 0.56 | 0.11 | 0.22 | 0.56 | 0.22 | 0.22 | 0.56 | 0.11 | | 0.11 |
| BN | 0.66 | 0.72 | 0.85 | 0.67 | 0.76 | 0.80 | 0.71 | 0.75 | 0.71 | 0.66 | 0.65 | | 0.68 |
| CF3 | 0.33 | 0.67 | 0.47 | 0.33 | 0.56 | 0.44 | 0.33 | 0.44 | 0.44 | 0.22 | 0.67 | | 0.22 |
| CF5 | 0.33 | 0.77 | 0.85 | 0.56 | 0.76 | 0.80 | 0.22 | 0.76 | 0.74 | 0.44 | 0.76 | | 0.73 |
|  | Subject9 | | | Subject10 | | | Subject11 | | | Subject12 | | | |
|  | LDA | ELM | SVM | LDA | ELM | SVM | LDA | ELM | SVM | LDA | ELM | SVM | |
| E | 0.33 | 0.44 | 0.44 | 0.22 | 0.44 | 0.44 | 0.33 | 0.33 | 0.44 | 0.22 | 0.33 | 0.44 | |
| PS | 0.44 | 0.44 | 0.33 | 0.33 | 0.22 | 0.33 | 0.22 | 0.56 | 0.33 | 0.56 | 0.22 | 0.33 | |
| WP | 0.44 | 0.22 | 0.33 | 0.44 | 0.44 | 0.33 | 0.44 | 0.22 | 0.33 | 0.44 | 0.22 | 0.33 | |
| TP | 0.33 | 0.22 | 0.33 | 0.22 | 0.33 | 0.11 | 0.22 | 0.11 | 0.33 | 0.33 | 0.11 | 0.44 | |
| BN | 0.65 | 0.67 | 0.71 | 0.56 | 0.61 | 0.58 | 0.45 | 0.48 | 0.47 | 0.73 | 0.81 | 0.65 | |
| CF3 | 0.33 | 0.44 | 0.22 | 0.22 | 0.44 | 0.44 | 0.33 | 0.33 | 0.22 | 0.56 | 0.56 | 0.11 | |
| CF5 | 0.44 | 0.67 | 0.73 | 0.44 | 0.67 | 0.63 | 0.44 | 0.67 | 0.63 | 0.60 | 0.84 | 0.72 | |
|  | Subject13 | | | Subject14 | | | Subject15 | | | Subject16 | | | |
|  | LDA | ELM | SVM | LDA | ELM | SVM | LDA | ELM | SVM | LDA | ELM | SVM | |
| E | 0.33 | 0.56 | 0.44 | 0.22 | 0.44 | 0.44 | 0.22 | 0.33 | 0.44 | 0.33 | 0.44 | 0.44 | |
| PS | 0.44 | 0.33 | 0.33 | 0.11 | 0.22 | 0.33 | 0.44 | 0.11 | 0.33 | 0.44 | 0.22 | 0.33 | |
| WP | 0.44 | 0.33 | 0.33 | 0.44 | 0.22 | 0.33 | 0.44 | 0.33 | 0.33 | 0.44 | 0.44 | 0.33 | |
| TP | 0.33 | 0.22 | 0.33 | 0.56 | 0.22 | 0.11 | 0.33 | 0.11 | 0.22 | 0.33 | 0.11 | 0.11 | |
| BN | 0.72 | 0.78 | 0.72 | 0.62 | 0.74 | 0.65 | 0.67 | 0.78 | 0.71 | 0.78 | 0.75 | 0.67 | |
| CF3 | 0.33 | 0.78 | 0.72 | 0.33 | 0.56 | 0.44 | 0.44 | 0.56 | 0.33 | 0.33 | 0.56 | 0.11 | |
| CF5 | 0.64 | 0.86 | 0.83 | 0.44 | 0.83 | 0.74 | 0.64 | 0.86 | 0.83 | 0.86 | 0.87 | 0.83 | |
|  | Subject17 | | | Subject18 | | | Subject19 | | | Subject20 | | | |
|  | LDA | ELM | SVM | LDA | ELM | SVM | LDA | ELM | SVM | LDA | ELM | SVM | |
| E | 0.22 | 0.33 | 0.44 | 0.33 | 0.33 | 0.44 | 0.11 | 0.33 | 0.44 | 0.44 | 0.67 | 0.33 | |
| PS | 0.44 | 0.11 | 0.33 | 0.22 | 0.22 | 0.33 | 0.22 | 0.56 | 0.33 | 0.33 | 0.11 | 0.44 | |
| WP | 0.44 | 0.22 | 0.33 | 0.44 | 0.33 | 0.33 | 0.44 | 0.33 | 0.33 | 0.33 | 0.22 | 0.22 | |
| TP | 0.67 | 0.67 | 0.44 | 0.44 | 0.22 | 0.22 | 0.56 | 0.33 | 0.33 | 0.22 | 0.44 | 0.44 | |
| BN | 0.82 | 0.85 | 0.85 | 0.76 | 0.78 | 0.78 | 0.56 | 0.64 | 0.76 | 0.65 | 0.67 | 0.65 | |
| CF3 | 0.22 | 0.33 | 0.33 | 0.44 | 0.67 | 0.22 | 0.11 | 0.67 | 0.22 | 0.56 | 0.44 | 0.44 | |
| CF5 | 0.56 | 0.94 | 0.93 | 0.82 | 0.96 | 0.93 | 0.62 | 0.74 | 0.83 | 0.73 | 0.74 | 0.73 | |

Table 7 Recognition results of three levels of the imagined hand clenching speeds by single trial for individual subject.

|  | Subject1 | | | Subject2 | | | Subject3 | | | Subject4 | | | | |
| --- | --- | --- | --- | --- | --- | --- | --- | --- | --- | --- | --- | --- | --- | --- |
|  | LDA | ELM | SVM | LDA | ELM | SVM | LDA | ELM | SVM | LDA | ELM | | SVM | |
| E | 0.33 | 0.22 | 0.56 | 0.22 | 0.11 | 0.56 | 0.33 | 0.11 | 0.56 | 0.33 | 0.33 | | 0.22 | |
| PS | 0.44 | 0.33 | 0.33 | 0.44 | 0.44 | 0.33 | 0.44 | 0.33 | 0.33 | 0.11 | 0.33 | | 0.33 | |
| WP | 0.44 | 0.67 | 0.33 | 0.33 | 0.56 | 0.33 | 0.44 | 0.33 | 0.33 | 0.44 | 0.33 | | 0.33 | |
| TP | 0.44 | 0.56 | 0.33 | 0.33 | 0.33 | 0.44 | 0.22 | 0.67 | 0.33 | 0.22 | 0.33 | | 0.56 | |
| BN | 0.64 | 0.75 | 0.78 | 0.72 | 0.78 | 0.83 | 0.76 | 0.82 | 0.85 | 0.64 | 0.78 | | 0.65 | |
| CF3 | 0.33 | 0.33 | 0.33 | 0.56 | 0.11 | 0.33 | 0.33 | 0.22 | 0.44 | 0.33 | 0.11 | | 0.33 | |
| CF5 | 0.76 | 0.81 | 0.84 | 0.74 | 0.82 | 0.83 | 0.44 | 0.93 | 0.93 | 0.64 | 0.82 | | 0.73 | |
|  | Subject5 | | | Subject6 | | | Subject7 | | | Subject8 | | | | |
|  | LDA | ELM | SVM | LDA | ELM | SVM | LDA | ELM | SVM | LDA | ELM | | SVM | |
| E | 0.33 | 0.11 | 0.22 | 0.44 | 0.11 | 0.56 | 0.33 | 0.22 | 0.56 | 0.33 | 0.33 | | 0.56 | |
| PS | 0.56 | 0.33 | 0.33 | 0.44 | 0.33 | 0.33 | 0.11 | 0.44 | 0.33 | 0.33 | 0.33 | | 0.44 | |
| WP | 0.44 | 0.56 | 0.33 | 0.33 | 0.56 | 0.33 | 0.44 | 0.56 | 0.33 | 0.33 | 0.56 | | 0.33 | |
| TP | 0.33 | 0.44 | 0.44 | 0.33 | 0.11 | 0.22 | 0.44 | 0.33 | 0.22 | 0.67 | 0.22 | | 0.22 | |
| BN | 0.56 | 0.54 | 0.54 | 0.66 | 0.69 | 0.67 | 0.66 | 0.76 | 0.68 | 0.68 | 0.75 | | 0.85 | |
| CF3 | 0.11 | 0.11 | 0.33 | 0.33 | 0.22 | 0.33 | 0.67 | 0.22 | 0.33 | 0.33 | 0.22 | | 0.56 | |
| CF5 | 0.63 | 0.71 | 0.63 | 0.74 | 0.73 | 0.73 | 0.74 | 0.82 | 0.73 | 0.71 | 0.82 | | 0.93 | |
|  | Subject9 | | | Subject10 | | | Subject11 | | | Subject12 | | | | |
|  | LDA | ELM | SVM | LDA | ELM | SVM | LDA | ELM | SVM | LDA | ELM | | SVM | |
| E | 0.33 | 0.33 | 0.22 | 0.56 | 0.33 | 0.56 | 0.33 | 0.11 | 0.56 | 0.33 | 0．22 | | 0.22 | |
| PS | 0.33 | 0.33 | 0.33 | 0.22 | 0.22 | 0.33 | 0.22 | 0.33 | 0.44 | 0.11 | 0.33 | | 0.33 | |
| WP | 0.44 | 0.11 | 0.33 | 0.44 | 0.22 | 0.33 | 0.33 | 0.56 | 0.33 | 0.44 | 0.56 | | 0.33 | |
| TP | 0.56 | 0.67 | 0.44 | 0.44 | 0.33 | 0.44 | 0.44 | 0.11 | 0.22 | 0.33 | 0.56 | | 0.44 | |
| BN | 0.65 | 0.78 | 0.83 | 0.56 | 0.64 | 0.67 | 0.56 | 0.67 | 0.68 | 0.75 | 0.67 | | 0.73 | |
| CF3 | 0.22 | 0.22 | 0.33 | 0.44 | 0.22 | 0.33 | 0.44 | 0.22 | 0.33 | 0.33 | 0.22 | | 0.33 | |
| CF5 | 0.72 | 0.81 | 0.87 | 0.62 | 0.67 | 0.73 | 0.63 | 0.72 | 0.68 | 0.76 | 0.71 | | 0.83 | |
|  | Subject13 | | | Subject14 | | | Subject15 | | | Subject16 | | | | |
|  | LDA | ELM | SVM | LDA | ELM | SVM | LDA | ELM | SVM | LDA | ELM | | SVM | |
| E | 0.44 | 0.11 | 0.56 | 0.22 | 0.22 | 0.22 | 0.56 | 0.22 | 0.22 | 0.33 | 0.11 | | 0.56 | |
| PS | 0.44 | 0.44 | 0.33 | 0.44 | 0.33 | 0.33 | 0.33 | 0.44 | 0.33 | 0.33 | 0.22 | | 0.44 | |
| WP | 0.33 | 0.44 | 0.33 | 0.44 | 0.33 | 0.33 | 0.44 | 0.44 | 0.33 | 0.33 | 0.33 | | 0.33 | |
| TP | 0.56 | 0.22 | 0.44 | 0.44 | 0.44 | 0.44 | 0.22 | 0.33 | 0.44 | 0.44 | 0.22 | | 0.33 | |
| BN | 0.65 | 0.78 | 0.82 | 0.74 | 0.68 | 0.75 | 0.74 | 0.65 | 0.78 | 0.67 | 0.78 | | 0.83 | |
| CF3 | 0.33 | 0.11 | 0.33 | 0.33 | 0.22 | 0.33 | 0.33 | 0.11 | 0.33 | 0.44 | 0.22 | | 0.33 | |
| CF5 | 0.74 | 0.81 | 0.84 | 0.83 | 0.81 | 0.83 | 0.81 | 0.78 | 0.83 | 0.72 | 0.83 | | 0.85 | |
|  | Subject17 | | | Subject18 | | | Subject19 | | | Subject20 | | | | |
|  | LDA | ELM | SVM | LDA | ELM | SVM | LDA | ELM | SVM | LDA | | ELM | | SVM |
| E | 0.33 | 0.11 | 0.22 | 0.33 | 0.11 | 0.56 | 0.33 | 0.22 | 0.22 | 0.56 | | 0.22 | | 0.56 |
| PS | 0.11 | 0.33 | 0.33 | 0.33 | 0.22 | 0.44 | 0.33 | 0.56 | 0.33 | 0.44 | | 0.33 | | 0.44 |
| WP | 0.44 | 0.44 | 0.33 | 0.33 | 0.67 | 0.33 | 0.44 | 0.44 | 0.33 | 0.33 | | 0.44 | | 0.33 |
| TP | 0.11 | 0.33 | 0.33 | 0.67 | 0.11 | 0.22 | 0.33 | 0.22 | 0.56 | 0.56 | | 0.11 | | 0.44 |
| BN | 0.69 | 0.75 | 0.72 | 0.73 | 0.83 | 0.81 | 0.56 | 0.65 | 0.67 | 0.78 | | 0.85 | | 0.89 |
| CF3 | 0.44 | 0.22 | 0.33 | 0.33 | 0.11 | 0.33 | 0.11 | 0.11 | 0.33 | 0.22 | | 0.22 | | 0.33 |
| CF5 | 0.76 | 0.82 | 0.83 | 0.84 | 0.92 | 0.93 | 0.64 | 0.72 | 0.73 | 0.82 | | 0.91 | | 0.93 |
