# Supplementary material for: Single-Trial Recognition of Imagined Forces and Speeds of Hand Clenching Based on Brain Topography and Brain Network
Source: Brain Topogr. 2018 Dec 31;32(2):240–54. doi: 10.1007/s10548-018-00696-3 (PMC6373301; doi:10.1007/s10548-018-00696-3)
Supplement: Supplementary file 2 — Supplementary material 2 (DOCX 27 KB) [file 10548_2018_696_MOESM2_ESM.docx]

The average correlation coefficients between the maps of all trials of all subjects and the typical topographical maps were calculated, shown in Tables 8~11.

Table 8 The average correlation coefficients between the maps of all trials of all subjects and the typical maps of actual hand clenching forces

|  | | the maps of all trials of all subjects | | | | | | | | |
| --- | --- | --- | --- | --- | --- | --- | --- | --- | --- | --- |
|  |  | 4kg-A | 4kg-B | 4kg-C | 10kg-A | 10kg-B | 10kg-C | 16kg-A | 16kg-B | 16kg-C |
| the typical maps | 4kg-A | **0.61** | 0.32 | 0.21 | 0.48 | 0.28 | 0.22 | 0.45 | 0.14 | 0.11 |
|  | 4kg-B | 0.32 | **0.59** | 0.27 | 0.33 | 0.44 | 0.20 | 0.19 | 0.45 | 0.09 |
|  | 4kg-C | 0.21 | 0.27 | **0.75** | 0.23 | 0.31 | 0.69 | 0.38 | 0.40 | 0.67 |
|  | 10kg-A | 0.48 | 0.33 | 0.23 | **0.65** | 0.26 | 0.33 | 0.57 | 0.21 | 0.16 |
|  | 10kg-B | 0.28 | 0.44 | 0.31 | 0.26 | **0.66** | 0.38 | 0.22 | 0.58 | 0.41 |
|  | 10kg-C | 0.22 | 0.20 | 0.69 | 0.33 | 0.38 | **0.73** | 0.13 | 0.25 | 0.70 |
|  | 16kg-A | 0.45 | 0.19 | 0.38 | 0.57 | 0.22 | 0.13 | **0.65** | 0.36 | 0.12 |
|  | 16kg-B | 0.14 | 0.45 | 0.40 | 0.21 | 0.58 | 0.25 | 0.36 | **0.71** | 0.40 |
|  | 16kg-C | 0.11 | 0.09 | 0.67 | 0.16 | 0.41 | 0.70 | 0.12 | 0.40 | **0.77** |

Table 9 The average correlation coefficients between the maps of all trials of all subjects and the typical maps of imagined hand clenching forces

|  | | the maps of all trials of all subjects | | | | | | | | |
| --- | --- | --- | --- | --- | --- | --- | --- | --- | --- | --- |
|  |  | 4kg-A | 4kg-B | 4kg-C | 10kg-A | 10kg-B | 10kg-C | 16kg-A | 16kg-B | 16kg-C |
| the typical maps | 4kg-A | **0.59** | 0.23 | 0.22 | 0.45 | 0.31 | 0.24 | 0.43 | 0.15 | 0.21 |
|  | 4kg-B | 0.23 | **0.62** | 0.24 | 0.26 | 0.40 | 0.21 | 0.17 | 0.43 | 0.12 |
|  | 4kg-C | 0.22 | 0.24 | **0.64** | 0.19 | 0.30 | 0.58 | 0.31 | 0.32 | 0.60 |
|  | 10kg-A | 0.45 | 0.26 | 0.19 | **0.60** | 0.27 | 0.25 | 0.52 | 0.31 | 0.17 |
|  | 10kg-B | 0.31 | 0.40 | 0.30 | 0.27 | **0.63** | 0.29 | 0.23 | 0.50 | 0.37 |
|  | 10kg-C | 0.24 | 0.21 | 0.58 | 0.25 | 0.29 | **0.64** | 0. 19 | 0.33 | 0.60 |
|  | 16kg-A | 0.43 | 0.17 | 0.31 | 0.52 | 0.23 | 0.19 | **0.65** | 0.38 | 0.32 |
|  | 16kg-B | 0.15 | 0.43 | 0.32 | 0.31 | 0.50 | 0.33 | 0.38 | **0.63** | 0.34 |
|  | 16kg-C | 0.21 | 0.12 | 0.60 | 0.17 | 0.37 | 0.60 | 0.32 | 0.34 | **0.69** |

Table10 The average correlation coefficients between the maps of all trials of all subjects and the typical maps of actual hand clenching speeds

|  | | the maps of all trials of all subjects | | | | | | | | |
| --- | --- | --- | --- | --- | --- | --- | --- | --- | --- | --- |
|  |  | 0.5Hz-A | 0.5Hz -B | 0.5Hz -C | 1Hz -A | 1Hz -B | 1Hz -C | 2Hz -A | 2Hz -B | 2Hz -C |
| the typical maps | 0.5Hz-A | **0.56** | 0.34 | 0.32 | 0.50 | 0.33 | 0.35 | 0.48 | 0.35 | 0.31 |
|  | 0.5Hz-B | 0.34 | **0.53** | 0.34 | 0.36 | 0.38 | 0.22 | 0.27 | 0.45 | 0.33 |
|  | 0.5Hz-C | 0.32 | 0.34 | **0.60** | 0.29 | 0.31 | 0.58 | 0.29 | 0.36 | 0.55 |
|  | 1Hz-A | 0.50 | 0.36 | 0.29 | **0.58** | 0.27 | 0.29 | 0.52 | 0.31 | 0.37 |
|  | 1Hz -B | 0.33 | 0.38 | 0.31 | 0.27 | **0.57** | 0.29 | 0.23 | 0.47 | 0.37 |
|  | 1Hz-C | 0.35 | 0.22 | 0.58 | 0.29 | 0.29 | **0.62** | 0. 28 | 0.35 | 0.47 |
|  | 2Hz-A | 0.48 | 0.27 | 0.29 | 0.52 | 0.23 | 0.28 | **0.56** | 0.37 | 0.33 |
|  | 2Hz-B | 0.35 | 0.45 | 0.36 | 0.31 | 0.47 | 0.35 | 0.37 | **0.63** | 0.40 |
|  | 2Hz-C | 0.31 | 0.33 | 0.55 | 0.37 | 0.37 | 0.47 | 0.33 | 0.40 | **0.64** |

Table11 The average correlation coefficients between the maps of all trials of all subjects and the typical maps of imagined hand clenching speeds

|  | | the maps of all trials of all subjects | | | | | | | | |
| --- | --- | --- | --- | --- | --- | --- | --- | --- | --- | --- |
|  |  | 0.5Hz-A | 0.5Hz -B | 0.5Hz -C | 1Hz -A | 1Hz -B | 1Hz -C | 2Hz -A | 2Hz -B | 2Hz -C |
| the typical maps | 0.5Hz-A | **0.51** | 0.35 | 0.38 | 0.44 | 0.33 | 0.37 | 0.46 | 0.27 | 0.39 |
|  | 0.5Hz-B | 0.35 | **0.57** | 0.27 | 0.35 | 0.41 | 0.34 | 0.37 | 0.49 | 0.29 |
|  | 0.5Hz-C | 0.38 | 0.27 | **0.55** | 0.31 | 0.32 | 0.48 | 0.39 | 0.34 | 0.43 |
|  | 1Hz-A | 0.44 | 0.35 | 0.31 | **0.53** | 0.37 | 0.31 | 0.47 | 0.33 | 0.38 |
|  | 1Hz -B | 0.33 | 0.41 | 0.32 | 0.37 | **0.59** | 0.28 | 0.33 | 0.42 | 0.31 |
|  | 1Hz-C | 0.37 | 0.34 | 0.48 | 0.31 | 0.30 | **0.58** | 0. 30 | 0.32 | 0.40 |
|  | 2Hz-A | 0.46 | 0.37 | 0.39 | 0.47 | 0.33 | 0.30 | **0.51** | 0.25 | 0.31 |
|  | 2Hz-B | 0.27 | 0.49 | 0.34 | 0.33 | 0.42 | 0.32 | 0.25 | **0.53** | 0.35 |
|  | 2Hz-C | 0.39 | 0.29 | 0.43 | 0.38 | 0.31 | 0.40 | 0.31 | 0.35 | **0.56** |

The permutation test for all five types features were used to determine chance level of the LDA, ELM and SVM, shown in Table12.

Table 12 The chance level of the LDA, ELM and SVM for all five types features

|  | Actual forces | | | Imagined forces | | | Actual speeds | | | Imagined speeds | | |
| --- | --- | --- | --- | --- | --- | --- | --- | --- | --- | --- | --- | --- |
|  | LDA | ELM | SVM | LDA | ELM | SVM | LDA | ELM | SVM | LDA | ELM | SVM |
| chance level | 33% | 34% | 35% | 34% | 33% | 36% | 33% | 32% | 35% | 32% | 35% | 36% |
